# Maximizing Bifunctionality for Overall Water Splitting by Integrating H_2_ Spillover and Oxygen Vacancies in CoPBO/Co_3_O_4_ Composite Catalyst

**DOI:** 10.1002/smsc.202400343

**Published:** 2024-11-10

**Authors:** Rinkoo Bhabal, Aniruddha Bhide, Suraj Gupta, Rohan Fernandes, Nainesh Patel

**Affiliations:** ^1^ Department of Physics and Electronics Christ University Bengaluru 560029 India; ^2^ Advanced Materials Department Jožef Stefan Institute Jamova 39 1000 Ljubljana Slovenia

**Keywords:** bifunctional, electrocatalysts, electrolysis, hydrogen spillover, oxygen vacancies

## Abstract

In the pursuit of utilizing renewable energy sources for green hydrogen (H_2_) production, alkaline water electrolysis has emerged as a key technology. To improve the reaction rates of overall water electrolysis and simplify electrode manufacturing, development of bifunctional electrocatalysts is of great relevance. Herein, CoPBO/Co_3_O_4_ is reported as a binary composite catalyst comprising amorphous (CoPBO) and crystalline (Co_3_O_4_) phases as a high‐performing bifunctional electrocatalyst for alkaline water electrolysis. Owing to the peculiar properties of CoPBO and Co_3_O_4_, such as complementing Gibbs free energy values for H‐adsorption (Δ*G*_H_) and relatively smaller difference in their work functions (ΔΦ), the composite exhibits H_2_ spillover (HS) mechanism to facilitate the hydrogen evolution reaction (HER). The outcome is manifested in the form of a low HER overpotential of 65 mV (at 10 mA cm^−2^). Moreover, an abundant amount of surface oxygen vacancies (O_v_) are observed in the same CoPBO/Co_3_O_4_ composite that facilitates oxygen evolution reaction (OER) as well, leading to a mere 270 mV OER overpotential (at 10 mA cm^−2^). The present work showcases the possibilities to strategically design non‐noble composite catalysts that combine the advantages of HS phenomenon as well as O_v_ to achieve new record performances in alkaline water electrolysis.

## Graphical abstract

The metal–organic framework‐derived CoPBO/Co_3_O_4_ electrocatalyst shows superior hydrogen evolution reaction activity due to phenomenon of hydrogen spillover from CoPBO to Co_3_O_4_, while the presence of abundant oxygen vacancies enhances the oxygen evolution reaction activity.

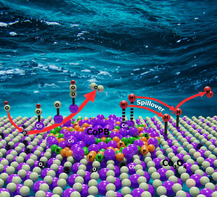

## 1 Introduction

Hydrogen (H_2_) is regarded as a promising alternative to fossil fuels due to its environment‐friendly and renewable attributes.^[^ ^1^ ^]^ Currently, around 95% of hydrogen is produced from fossil fuels through reforming or gasification, known as “grey hydrogen,” costing about 1–2 USD kg^−1^. However, the complex purification of grey hydrogen generates about 10 kg of CO_2_ emissions per kg of hydrogen produced.^[^ ^2^ ^]^ Compared to traditional techniques, electrochemical water splitting stands out as one of the most sustainable and carbon‐neutral methods for green hydrogen production, free from any hazardous by‐products.^[^ ^3^ ^]^ Green hydrogen is expected to replace about 30% of grey hydrogen by 2030. Despite this, its production cost is still roughly 4–5 times higher than that of grey hydrogen.^[^ ^2^ ^]^ A key bottleneck in upscaling this technology is the inability to find the right balance between the cost, stability, and efficiency of the critical components of a water electrolyzer, such as the electrocatalyst and membrane. A commercially viable electrocatalyst demands exceptional performance for both hydrogen evolution reaction (HER) and oxygen evolution reaction (OER). To meet such demands, bifunctional electrocatalysts are used that can effectively catalyze the production of hydrogen while simultaneously facilitating the generation of oxygen with high efficiency and stability.

HER and OER are fundamentally different reactions, and hence, the bifunctional electrocatalysts must possess attributes that are suitable to facilitate both reactions. For instance, the electrocatalytic HER is governed by the principles of the volcano theory, wherein an electrocatalyst with an optimum Gibbs free energy of adsorption (Δ*G*_H _≈ 0) is considered ideal for HER activity.^[^ ^1^, ^4^ ^]^ Therefore, a good HER electrocatalyst must be one that has Δ*G*_H_ value close to the top of the volcano, as seen in electrocatalysts based on the platinum group metals (PGMs).^[^ ^1^ ^]^ However, the high cost and limited availability of PGMs hinder their widespread adoption for large‐scale applications. In response, electrocatalysts derived from non‐noble metals (e.g., Co, Ni, Fe, Mo) in the form of transition metal borides,^[^ ^1^ ^]^ phosphides,^[^ ^5^ ^]^ sulfides,^[^ ^6^ ^]^ and selenides^[^ ^7^ ^]^ have been identified as highly efficient options for water electrolysis. Likewise, in binary composite electrocatalysts, hydrogen spillover (HS), observed at the interface between a metal and support material, is identified as a promising phenomenon to achieve unprecedented HER activities. Such binary catalysts involve strong proton adsorption on metal (Δ*G*_H‐metal_ < 0), which spill over to support (Δ*G*_H‐support_ > 0)^[^ ^8^, ^9^ ^]^ for efficient hydrogen desorption. Moreover, it offers a cost‐effective solution by reducing the need for precious metals by distributing the catalytic load between the metal and the support. However, a major obstacle for interfacial hydrogen transfer may arise due to the energy barrier resulting from the substantial difference in work function (ΔΦ) between the metal and the support. The Schottky barrier generated due to the large difference between the Fermi energies of metal and support leads to charge accumulation and, consequently, strong proton trapping at the interface.^[^ ^8^, ^9^, ^10^ ^]^ In this scenario, the process of HS from metal to support encounters a substantial energy barrier, resulting in diminished HER performance. Conversely, when the metal and support exhibit similar Fermi energies, the formation of the Schottky barrier will be restrained and minimize the interfacial charge accumulation. Thus, to enhance the HER activity, it is essential to create a binary metal/support electrocatalyst with minimal difference in work function (ΔΦ).^[^ ^8^, ^9^, ^10^, ^11^, ^12^, ^13^, ^14^ ^]^ While a majority of the studied binary composite catalysts demonstrating HS are constructed using PGMs,^[^ ^8^, ^9^, ^10^ ^]^ there are a limited number of reports where non‐PGM^[^ ^13^ ^]^ catalysts are used. Thus, there is a huge opportunity to explore the phenomenon of HS in non‐PGM catalysts like transition metals and achieve superior HER activities, as achieved in this work.

Unlike HER, OER is a kinetically sluggish reaction due to the multi‐electron transfer steps involved. Noble metal oxides like IrO_2_ and RuO_2_ have been reported as active and stable electrocatalysts for OER but are undesired because of their high cost and scarcity.^[^ ^15^, ^16^, ^17^ ^]^ Transition metal oxide (TMO)‐derived electrocatalysts, such as oxyhydroxides,^[^ ^18^, ^19^ ^]^ perovskites,^[^ ^20^, ^21^ ^]^ layered double hydroxides, and spinels,^[^ ^22^, ^23^ ^]^ are often reported as non‐noble alternatives for OER. Among these, metal oxides such as spinel cobalt oxide (Co_3_O_4_) show considerable OER performance and stability. To boost the intrinsic performance of such metal oxides, defect engineering, such as forming surface oxygen vacancies (O_v_), has emerged as an effective strategy.^[^ ^18^, ^19^, ^20^, ^22^, ^23^, ^24^, ^25^, ^26^, ^27^, ^28^ ^]^ Some of the key methods to create oxygen vacancies include ionic doping, bombardment of the surface with high‐energy particles, high‐temperature treatment, and chemical reduction methods. Among them, the chemical reduction method stands out as a promising methodology due to its simplicity and versatility.^[^ ^29^ ^]^ Oxygen vacancies play a crucial role in regulating the electronic structure by reducing the coordination number of active metal sites, which enhances the adsorption of reactant ions and facilitates OER.^[^ ^29^ ^]^

Indeed, achieving optimal bifunctional activity necessitates incorporating both the HS phenomenon and the oxygen vacancies within the same electrocatalyst system. The synergistic interplay between these two factors will prove crucial in attaining superior bifunctional capabilities within the electrocatalyst. However, to the best of our knowledge, no reported electrocatalyst composite has demonstrated both of these phenomena concurrently. The present work reports a nanocomposite comprising amorphous cobalt phospho‐borate (CoPBO) and crystalline cobalt oxide (Co_3_O_4_), showcasing both the HS mechanism and a substantial quantity of oxygen vacancies, resulting in exceptional bifunctional activity for water splitting. To elucidate the role of each component in the composite, we conducted in situ kinetic studies employing electrochemical impedance spectroscopy (EIS) and cyclic voltammetry (CV) to probe the reaction kinetics of the HER and OER. This discovery of a novel electrocatalyst composite opens possibilities for a new category of non‐noble bifunctional electrocatalysts and could offer valuable insights into enhancing the efficiency of alkaline water electrolysis systems.

## 2 Results and Discussion

Cobalt metal–organic framework (Co‐MOF) was initially prepared via a solvothermal method and subsequently pyrolyzed at 700 °C in air, leading to its conversion into Co_3_O_4_. This MOF‐derived Co_3_O_4_ and as‐prepared Co‐MOF were further chemically reduced using NaBH_4_ and NaH_2_PO_2_ that also act as the B and P sources, respectively, to form cobalt phospho‐borate (CoPBO), ultimately yielding MOF‐derived CoPBO/Co_3_O_4_ and CoPBO@Co‐MOF, respectively (Scheme S1, Supporting Information).

**Figure 1:**
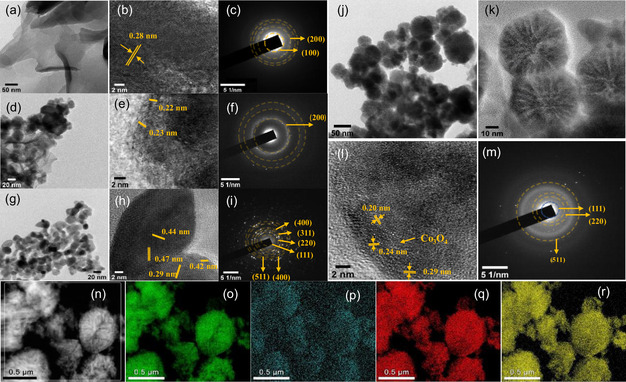
a,d,g,j,k) TEM images, b,e,h,l) HRTEM images, c,f,i,m) SAED pattern of Co‐MOF, CoPBO@Co‐MOF, MOF‐derived Co_3_O_4_, and CoPBO/Co_3_O_4_, respectively. n) Reference HAADF image of CoPBO/Co_3_O_4_ for elemental mapping of o) Co, p) B, q) P, and r) O.

Electron microscopy reveals the intriguing morphological and structural transformations in the prepared catalysts. Scanning electron microscope (SEM) images (Figure S1a–c, Supporting Information) of Co‐MOF confirmed a nanosheet morphology with a smooth surface and a thickness of ≈30 nm (Figure S1a, Supporting Information). These nanosheets exhibit random entanglement, resulting in the formation of a 3D hierarchical and porous structure (Figure S1b,c, Supporting Information). Similar morphological features were observed in transmission electron microscope (TEM) micrographs (**Figure** **1**a and S2a, Supporting Information). High‐resolution TEM (HRTEM) revealed that the Co‐MOF possessed a crystalline nature with distinct lattice fringes (0.28 nm) (Figure 1b) and also corresponding diffraction rings of (100) and (200) planes in the selective area electron diffraction (SAED) pattern (Figure 1c). The SEM (Figure S1d, Supporting Information) and TEM (Figure 1d) images of CoPBO@Co‐MOF reveal that the chemical reduction process grafts the particles onto the surface of Co‐MOF nanosheets. While the underlying Co‐MOF nanosheets maintain their crystallinity with partially visible lattice fringes (Figure 1e) and diffraction rings (Figure 1f), the surface particles predominantly appear in an amorphous state, probably associated with the CoPBO compound (Figure S2c, Supporting Information). Such amorphous particles were also detected during the formation of the CoPBO powder catalyst by the chemical reduction of cobalt salt.^[^ ^30^ ^]^ After pyrolysis at 700 °C, the morphology of Co‐MOF undergoes a complete transformation from 2D nanosheets to particle‐like morphology with an average diameter of ≈22 nm (Figure 1g and S1e, Supporting Information). A highly crystalline structure was observed with distinct lattice planes (Figure 1h) and diffraction rings corresponding to the spinel structure of Co_3_O_4_ (Figure 1i).^[^ ^31^ ^]^ This particle‐like morphology was retained after chemical reduction in the case of CoPBO/Co_3_O_4_, although there was a significant increase in particle size, with an average diameter of ≈53 nm (Figure 1j and S1f, Supporting Information). However, different zones of variable contrast are visible in each of these particles (Figure 1k and S2b, Supporting Information). Further magnification in the HRTEM image shows that the lighter contrast zone is composed of distinct fringes with interplanar spacing assigned to the planes of the Co_3_O_4_ phase, while the darker region does not show any sign of crystallinity. The existence of this mixture of amorphous and crystalline phases in the particles is confirmed by the fast Fourier transform (FFT) and inverse FFT images for CoPBO@Co‐MOF (Figure S2c, Supporting Information) and CoPBO/Co_3_O_4_ (Figure S2d, Supporting Information). Also, in SAED, very few diffraction spots in the ring pertinent to Co_3_O_4_, along with a diffuse ring associated with the amorphous phase, are visible in CoPBO/Co_3_O_4_. This finding depicts that the incorporation of B and P induces the enlargement of the particles and forming zones containing crystalline Co_3_O_4_ and amorphous zones, which might be the indication of the formation of CoPBO. Such mixed‐phase CoPBO/Co_3_O_4_ composites are anticipated to be advantageous for catalytic reactions due to the unique properties provided by their complementary functions. The uniform distribution of Co, P, B, and O atoms was verified by the energy‐dispersive X‐ray analysis elemental mapping of CoPBO/Co_3_O_4_ from scanning transmission electron microscopy‐high‐angle annular dark‐field (HAADF) image (Figure 1n–r) and SEM image (Figure S3, Supporting Information).

**Figure 2:**
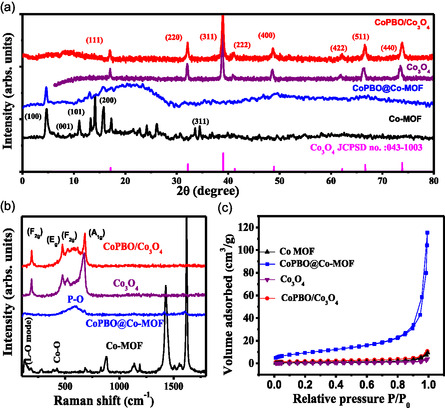
a) XRD pattern, b) Raman spectra, and c) N_2_ adsorption–desorption isotherm of Co‐MOF, CoPBO@Co‐MOF, MOF‐derived Co_3_O_4_, and CoPBO/Co_3_O_4_.

X‐ray diffraction (XRD) pattern (**Figure** **2**a) exhibits prominent diffraction peaks at 8.8°, 13.96°, 15.76°, 17.88°, and 33.06° corresponding to the respective crystallographic planes (100), (001), (101), (200), and (311) of Co‐MOF. The XRD pattern of as‐synthesized Co‐MOF matches well with the previous report on Co‐MOF.^[^ ^32^ ^]^ After the phospho‐boronization process, there is a noticeable reduction in the intensity of these diffraction peaks of Co‐MOF. Nevertheless, the (100) peak remains detectable even after the treatment, indicating that Co‐MOF partially retains its structure within CoPBO@Co‐MOF. A broad signal at higher 2*θ* values suggests surface coverage with amorphous CoPBO particles, as also observed from HRTEM image (Figure 1g). After the controlled pyrolysis treatment of Co‐MOF at 700 °C, several new peaks appear at 18.9°, 31.3°, 36.7°, 44.6°, 55.5°, 59.2°, and 64.9° that are assigned to (111), (220), (311), (400), (422), (511), and (440) planes, respectively, of spinel Co_3_O_4_^[^ ^33^ ^]^ (JCPDS no. 043‐1003). This confirms the complete transformation of Co‐MOF to pure phase spinel Co_3_O_4_. In CoPBO/Co_3_O_4_ sample, no significant alterations are observed in the diffraction pattern, indicating the preservation of the crystalline structure of Co_3_O_4_ even after the phospho‐boronization treatment. Raman spectroscopy was employed to investigate the surface structural changes of electrocatalysts (Figure 2b). In the Co‐MOF sample, characteristic peaks at 131 and 424 cm^−1^ are associated with the longitudinal mode and Co–O vibrations, respectively. Concurrently, bands around 871, 1135, 1425, and 1620 cm^−1^ are attributed to the organic benzene‐1,4‐dicarboxylate (BDC) linkers.^[^ ^34^, ^35^, ^36^ ^]^ None of these peaks are discernible in the CoPBO@Co‐MOF sample, and instead, a broad peak ranging from 500 to 650 cm^−1^ is attributed to stretching vibrations associated with the O–P–O bending mode^[^ ^37^ ^]^ within the CoPBO structure. This further affirms the surface coverage with amorphous CoPBO particles. Upon pyrolysis of Co‐MOF, distinctive peaks corresponding to the *F*_2g_ (184 cm^−1^), *E*_g_ (474 cm^−1^), *F*_2g_ (529 cm^−1^), and *A*_1g_ (676 cm^−1^) vibration modes of Co_3_O_4_ emerge.^[^ ^38^, ^39^ ^]^ However, after the phospho‐boronization treatment in CoPBO/Co_3_O_4_, the intensity of these peaks diminishes, accompanied by the appearance of a broad feature within the 500–650 cm^−1^ range, resembling the characteristics of CoPBO. This observation aligns with the findings from the HRTEM, XRD, and SAED patterns, collectively confirming the formation of a composite structure consisting of crystalline Co_3_O_4_ and amorphous CoPBO composite. The physical surface area, as determined by Brunauer–Emmett–Teller (BET) analysis (Figure 2c), of Co‐MOF, CoPBO@Co‐MOF, MOF‐derived Co_3_O_4_, and CoPBO/Co_3_O_4_ is 3.2, 34.2, 1.6, and 6.5 m^2^ g^−1^, respectively. These values depict that although the surface area reduces after calcination, the phospho‐boronization process improves the surface area, which may assist in surface redox reactions.

**Figure 3:**
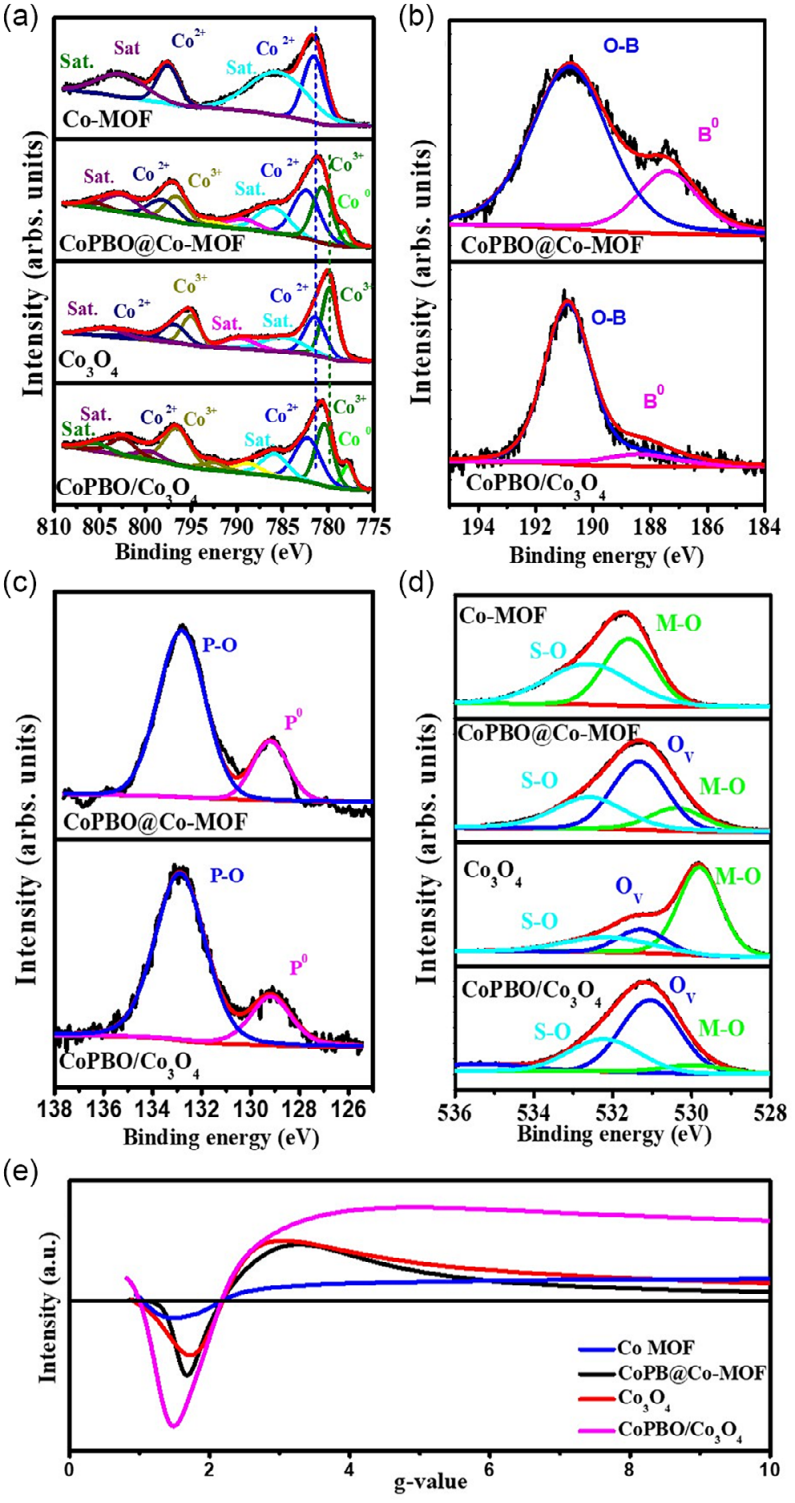
XPS spectra of a) Co 2*p*, b) B 1*s*, c) P 2*p*, and d) O 1*s* levels, and e) ESR spectra of Co‐MOF, CoPBO@Co‐MOF, MOF‐derived Co_3_O_4_, and CoPBO/Co_3_O_4_.

High‐resolution X‐ray photoelectron spectroscopy (XPS) analysis of the Co 2*p*_3/2_ region (**Figure** **3**a) in Co‐MOF revealed two distinct peaks centered at 781.5 and 785.8 eV, corresponding to the Co^2+^ state and its corresponding satellite, respectively.^[^ ^40^, ^41^ ^]^ Upon pyrolysis at 700 °C, both Co^3+^ and Co^2+^ states are evident at peak positions of 779.7 and 781.5 eV, along with their related satellite peaks.^[^ ^42^, ^43^ ^]^ The ratio of the peak area for Co^2+^ to Co^3+^ confirmed the formation of a pure spinel Co_3_O_4_ phase. After phospho‐boronization of both Co‐MOF and Co_3_O_4_, an additional metallic peak of cobalt emerges at 778.1 eV in CoPBO@Co‐MOF and at 777.8 eV in CoPBO/Co_3_O_4_. The Co^3+^ (780.6–780.5 eV) and Co^2+^ (782.3–782.2 eV) peaks, along with their respective shakeup peaks, are shifted positively in comparison with Co_3_O_4_ and are aligned with those reported for CoPBO powder,^[^ ^30^, ^44^ ^]^ thus confirming the formation of CoPBO on the surface. Boron (B 1*s*) was detected in the form of oxides (191.0 eV) with a minor elemental component at 187.4 eV for CoPBO@Co‐MOF (Figure 3b). The elemental boron peaks were positively shifted compared to pure boron (187.1 eV).^[^ ^30^, ^44^, ^45^, ^46^ ^]^ In contrast, for CoPBO/Co_3_O_4_, boron was predominantly present in an oxidized state. Similarly, phosphorus was also found in both oxidized (132.8 and 132.7 eV) and elemental (129.2 and 129.1 eV) forms for the catalysts containing CoPBO (Figure 3c). The binding energy of the elemental peak was negatively shifted in comparison with pure phosphorus (130.2 eV), implying electron enrichment over phosphorus atoms and electron deficiency over boron atoms.^[^ ^30^, ^47^ ^]^ This electron modulation between boron and phosphorus through transition metals is a unique feature of the phospho‐borate catalyst.^[^ ^30^ ^]^ In the O 1*s* region (Figure 3d), two distinct peaks at 530.7 and 532.6 eV were deconvoluted and assigned to the metal–oxygen bond (M–O) and surface–adsorbed oxygen (S–O), respectively, for Co‐MOF.^[^ ^48^ ^]^ After the conversion to Co_3_O_4_, lattice oxygen with a primary peak at 529.8 eV was visible, along with a new minor peak at 531.3 eV.^[^ ^26^, ^28^, ^29^ ^]^ This new signal was recently distinguished for the presence of oxygen in the hydroxyl group linked to the unoccupied oxygen vacancy sites (O_v_). According to Idris et al.^[^ ^49^ ^]^ and also supported by a dedicated article on XPS interpretation of oxygen vacancies,^[^ ^50^ ^]^ it is important to note that the peak observed at 531.5 eV does not directly signify O_v_. Instead, it indicates the existence of OH species. This is because oxygen defects on the surface are inherently unstable and are promptly replaced by OH species during the process of water's dissociative adsorption. Therefore, while the 531.5 eV peak is attributed to adsorbed OH species, it is indirectly associated with O_v_ or defects on the surface.^[^ ^28^ ^]^ After the reduction reaction, the peak corresponding to O_v_ (531.3–531.5 eV) grows stronger in CoPBO@Co‐MOF and CoPBO/Co_3_O_4_, indicating surface reduction during phospho‐boronization. The O_v_/M–O peak ratios of 2.89, 0.29, and 8.21 for CoPBO@Co‐MOF, MOF‐derived Co_3_O_4_, and CoPBO/Co_3_O_4_, respectively, advocate the formation of a substantial amount of oxygen vacancies^[^ ^28^ ^]^ during the phospho‐boronization process, with the largest amount observed in CoPBO/Co_3_O_4_ composite (Table S1, Supporting Information). In addition, electron spin resonance (ESR) was utilized to confirm and quantify the presence of O_v_. ESR analysis can detect the electrons in an unpaired state generated due to oxygen vacancy formation, thus indirectly indicating their presence. CoPBO/Co_3_O_4_, along with control electrocatalysts, showed the ESR signal at a *g*‐value of 2.1, confirming the presence of O_v_.^[^ ^51^ ^]^ Also, CoPBO/Co_3_O_4_ showed the highest intensity of the ESR signal compared to Co_3_O_4_ and CoPB@Co‐MOF, while pristine Co‐MOF showed the lowest signal intensity. Thus, ESR analysis confirms the formation of a large number of O_v_ in CoPBO/Co_3_O_4_, supporting the XPS results.

**Figure 4:**
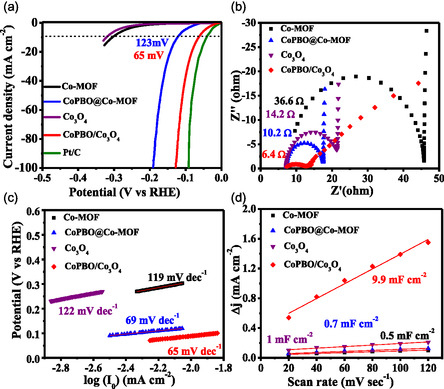
a) Linear polarization curves, b) electrochemical impedance spectra, c) Tafel plot for HER, and d) plot representing the differences in cathodic and anodic current densities at different scan rates to determine the *C*_dl_ values of Co‐MOF, CoPBO@Co‐MOF, MOF‐derived Co_3_O_4_, and CoPBO/Co_3_O_4_ recorded in 1 M KOH.

The HER performance of the optimized catalysts was investigated in a three‐electrode‐based electrochemical cell containing 1 M KOH. Based on the linear polarization data, CoPBO/Co_3_O_4_ required the lowest overpotential of 65 mV to achieve the benchmark current density of 10 mA cm^−2^, followed by CoPBO@Co‐MOF (123 mV), MOF‐derived Co_3_O_4_ (319 mV), and Co‐MOF (410 mV). The obtained overpotential for CoPBO/Co_3_O_4_ is a mere 28 mV higher than the Pt/C catalyst (39 mV), which implies that the composite structure is highly favorable for HER. It is worth noting that pristine CoPBO, synthesized via the conventional chemical reduction route, exhibits a higher overpotential of 145 mV under similar conditions (Figure S4a, Supporting Information), emphasizing the promoting role played by the formation of the CoPBO/Co_3_O_4_ and CoPBO@Co‐MOF composite. The effectiveness of these combinations in composite catalysts was further substantiated by testing the control samples. The CoBO@Co‐MOF and CoPO@Co‐MOF, fabricated by a similar method as CoPBO@Co‐MOF, were inactive for HER (Figure S5, Supporting Information). Additionally, CoBO/Co_3_O_4_ and CoPO/Co_3_O_4_ prepared using a similar approach as that of CoPBO/Co_3_O_4_, also required considerably higher potentials of 151 and 168 mV, respectively (Figure S6a, Supporting Information), thus highlighting the promoting role played by the blend of P and B. A reference CoPBO@Co_3_O_4_ catalyst was also synthesized, where CoPBO is formed over Co_3_O_4_ particles fabricated by a conventional coprecipitation route (see characterization and electrochemical results in ESI, Figure S7, Supporting Information). Despite similar morphology and particle size (≈50 nm) as that of MOF‐derived CoPBO/Co_3_O_4_, CoPBO@Co_3_O_4_ exhibited a much higher HER overpotential of 175 mV@10 mA cm^−2^ (Figure S4a, Supporting Information). These control tests highlight the importance of the porous framework produced by MOF, which promotes the formation of a mixed phase of amorphous CoPBO and crystalline Co_3_O_4_ within the particles, in contrast to the surface coverage of CoPBO over Co_3_O_4_ particles as in CoPBO@Co_3_O_4_. Upon comparing the overpotential values of recently reported electrocatalysts, CoPBO/Co_3_O_4_ stands out as one of the better electrocatalysts for HER (Table S2, Supporting Information). The presence of this mixed phase also contributes toward the reduction of charge transfer resistance (*R*_CT_) at the catalyst/electrolyte interface for CoPBO/Co_3_O_4_ (6.4 Ω) compared to other counterparts (**Figure** **4**b and S4b, S5b, S6b, Supporting Information). Furthermore, the Tafel slope value further endorses the superior HER kinetics of CoPBO/Co_3_O_4_ with the lowest slope value of 65 mV dec^−1^ (Figure 4c and S4c, S5c, S6c, Supporting Information). Most importantly, the double layer capacitance (*C*_dl_) values (Figure 4d and S8, Supporting Information) for MOF‐derived CoPBO/Co_3_O_4_ (9.9 mF cm^−2^) were about one order of magnitude higher than the Co‐MOF (0.5 mF cm^−2^), CoPBO@Co‐MOF (0.7 mF cm^−2^), and MOF‐derived Co_3_O_4_ (1.0 mF cm^−2^), which indicates the presence of a substantial number of active sites on the catalyst surface. Notably, the intrinsic activity of these sites is also high, as evidenced by the turnover frequency (TOF) values (@150 mV overpotential) for CoPBO/Co_3_O_4_ (0.049 atom^−1 ^s^−1^), which significantly surpasses that of CoPBO@Co‐MOF (0.005 atom^−1 ^s^−1^) and other catalysts. The intrinsic activity was also confirmed by BET surface area and electrochemical surface area (ECSA) normalized linear sweep voltammetry (LSV) curves (Figure S9, Supporting Information). To ensure that the HER data remain unaffected by Pt contamination from the counter electrode, the LSV data were replicated using graphite as the counter electrode. The obtained results were consistent and reproducible (Figure S10, Supporting Information).

As stated before, the HS phenomenon has surfaced as a promoting factor for augmenting the HER in binary composite catalysts.^[^ ^9^, ^10^, ^11^ ^]^ Thus, further investigations are focused on exploring the HS in the present CoPBO/Co_3_O_4_ composite catalyst. For HS mechanism, three main criteria must be fulfilled: 1) one of the two materials should have negative Gibbs free energy for H‐adsorption (Δ*G*_H_); 2) another material should have positive Δ*G*_H_ to facilitate the H‐desorption; and finally, and 3) the difference in work function (ΔΦ) between the two materials must be minimum to avoid charge accumulation at the interface.^[^ ^11^ ^]^ The hydrogen adsorption energy for the amorphous CoPBO catalyst, as calculated in our previous work using density functional theory (DFT) calculation, is −0.22 eV,^[^ ^30^ ^]^ while that for crystalline Co_3_O_4_ is around +0.2 eV.^[^ ^52^ ^]^ These values definitely meet the first two criteria for HS. The work function of 5.27 eV was calculated for the amorphous CoPBO cluster (see ESI for the calculation), while that for crystalline Co_3_O_4_, 5.35 eV is reported.^[^ ^53^ ^]^ Based on these values, the ΔΦ for CoPBO/Co_3_O_4_ binary catalyst is 0.08 eV, suggesting it to be an ideal composite candidate to demonstrate HS.

**Figure 5:**
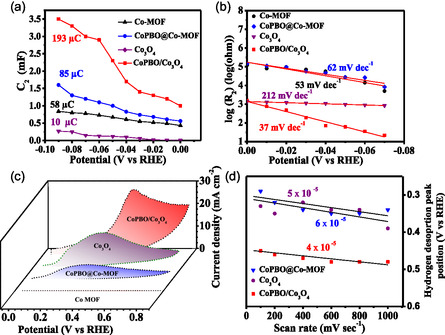
a) Plot of C_2_ versus applied potential, b) EIS‐derived Tafel plots, c) CV at the scan rate of 50 mV s^−1^, and d) plots of hydrogen desorption peak position versus scan rate of Co‐MOF, CoPBO@Co‐MOF, MOF‐derived Co_3_O_4_, and CoPBO/Co_3_O_4_ recorded for HER in 1 M KOH.

Further experiments were performed to study the hydrogen adsorption and desorption kinetics and verify the proposed HS phenomenon as the main reason behind enhanced HER in the present catalyst.^[^ ^11^ ^]^ To investigate the hydrogen adsorption behavior, EIS was carried out on Co‐MOF, CoPBO@Co‐MOF, MOF‐derived Co_3_O_4_, and CoPBO/Co_3_O_4_ at different potential values. All the recorded Nyquist plots were fitted by a double parallel equivalent circuit (Figure S11, Supporting Information)^[^ ^11^, ^15^ ^]^ where the first parallel circuit is related to the charge transfer kinetics, in which *C*_1_ represents the double layer capacitance and *R*_1_ represents the charge transfer resistance. On the other hand, the second parallel circuit corresponds to the hydrogen adsorption behavior on the catalyst surface, with components *C*_2_ and *R*_2_ representing hydrogen adsorption pseudocapacitance and resistance, respectively. The estimated *R*_1_ value for CoPBO/Co_3_O_4_ is the lowest, with no significant variation with *E*, suggesting fast charge transfer kinetics for HER (Table S3, Supporting Information). The hydrogen adsorption charge (*Q*_H_) on the surface of the electrocatalyst during HER was deduced by integrating the plot of *C*_2_ versus potential (**Figure** **5**a and S12a, S13a, Supporting Information). CoPBO/Co_3_O_4_ displayed highest *Q*_H_ value of 193 μC followed by CoPBO@Co‐MOF (85 μC), CoPBO@Co_3_O_4_ (72 μC), Co‐MOF (58 μC), CoPBO (24 μC), CoBO@Co‐MOF (17 μC), and MOF‐derived Co_3_O_4_ (10 μC). The *Q*_H_ values imply an improvement in H‐adsorption due to the presence of CoPBO, especially in CoPBO/Co_3_O_4_ composite. The hydrogen adsorption can be further quantified by assessing the Tafel slope value obtained by linear fitting the data in the plot of log(*R*_2_) versus potential. The low value of the EIS‐derived Tafel slope advocates accelerated hydrogen adsorption kinetics. As depicted in Figure 5b and S12b, S13b, Supporting Information, the EIS‐derived Tafel slope was found to be the lowest for CoPBO/Co_3_O_4_ (37 mV dec^−1^) among all catalysts, which is significantly lower than that of MOF‐derived Co_3_O_4_ (212 mV dec^−1^). These results reveal that the H‐adsorption characteristics of intrinsically inactive Co_3_O_4_ were significantly improved by forming a composite with CoPBO, resulting in profoundly enhanced HS from the latter to the former.

To investigate the hydrogen desorption kinetics, CVs were measured in the double‐layer region with different scan rates (Figure S14, Supporting Information).^[^ ^9^, ^10^, ^11^ ^]^ At the scan rate of 50 mV s^−1^, the hydrogen desorption peak is clearly evident for the Co_3_O_4_‐based catalyst, with the CoPBO/Co_3_O_4_ binary catalyst showcasing the highest peak intensity (Figure 5c and S12c, S13c, Supporting Information). On the contrary, the CoPBO@Co‐MOF displayed minimum intensity, while no such peak was detected in Co‐MOF. The above facts illustrate that Co_3_O_4_, which is inactive for H‐adsorption, is, in fact, ideal for hydrogen desorption. Upon forming a composite with CoPBO, the desorption rate increases with an abundant amount of HS from CoPBO to Co_3_O_4_ in CoPBO/Co_3_O_4_ composite. This finding was further supported by monitoring the shift in hydrogen desorption peak with scan rate. With increasing scan rate, the H_2_ desorption peak shifts to a higher potential due to the response time taken to reach the current at the applied potential. Thus, a lower shift in the peak position indicates faster desorption kinetics on the surface. This can be quantified from the slope values obtained by linearly fitting the plot of hydrogen desorption peak position versus scan rate (Figure 5d and S12d, S13d, Supporting Information). The lowest slope value of CoPBO/Co_3_O_4_ (4 × 10^−5^) compared to CoPBO@Co‐MOF (6 × 10^−5^), MOF‐derived Co_3_O_4_ (5 × 10^−5^), CoBO@Co‐MOF (6 × 10^−5^), CoPBO (20 × 10^−5^), and CoPBO@Co_3_O_4_ (5 × 10^−5^) reflects the accelerated hydrogen desorption kinetics in CoPBO/Co_3_O_4_ binary composite. The empirical evidence provided here clearly indicates the occurrence of the HS phenomenon in the CoPBO/Co_3_O_4_ binary composite catalyst. The rationale behind this phenomenon lies in enhanced hydrogen adsorption and desorption kinetics, coupled with minimal charge accumulation at the interface owing to ΔΦ, facilitating effective H‐spillover from CoPBO to Co_3_O_4_. Such an HS mechanism is also partially observed in the CoPBO@Co‐MOF sample, which showed the second‐best HER activity. The reported work function of Co‐MOF (5.0 eV) is very close to CoPBO (5.27 eV) with ΔΦ = 0.27 eV, but it is slightly higher than that of CoPBO/Co_3_O_4_ composite. Also, the desorption kinetics over Co‐MOF is negligible compared to Co_3_O_4_. Thus, the HER overpotential of 123 mV in this catalyst is higher than CoPBO/Co_3_O_4_ but lower than the pristine CoPBO catalyst (145 mV).

Further, the surface of the electrocatalyst post‐HER (after a 12 h chronoamperometric test) was probed with various techniques. In XPS spectra (Figure S15, Supporting Information) of CoPBO/Co_3_O_4_ post‐HER, the metallic peak of Co completely disappears while the peak position pertaining to Co^2+^ and Co^3+^ is maintained with a minor reduction in the intensity of the Co^2+^ peak. Similarly, the peaks corresponding to oxidized phosphorous and boron are retained, while the elemental peaks vanished from the spectra. In the Raman spectra (Figure S16, Supporting Information), the broad peak associated with O–P–O vibrations disappear post‐HER, while Co–O modes (*E*_g_, *F*_2g_, and *A*_1g_) are preserved. The morphological analysis further reveals that spherical particles are agglomerated, forming a large cluster after HER (Figure S17, Supporting Information). All these techniques provided physical evidence that the nature of the CoPBO/Co_3_O_4_ surface is altered after HER. Nevertheless, the bulk structure of the catalyst remains intact post‐HER, as verified by the XRD pattern (Figure S18, Supporting Information).

**Figure 6:**
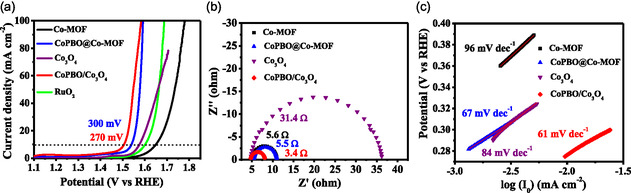
a) Linear polarization curves, b) electrochemical impedance spectra, and c) Tafel plot for OER of Co‐MOF, CoPBO@Co‐MOF, MOF‐derived Co_3_O_4_, and CoPBO/Co_3_O_4_ recorded in 1 M KOH.

The HS mechanism assists in improving the HER rate, but there are different factors that may assist in improving the OER rates. To assess the OER activity, LSVs were recorded in the anodic region (**Figure** **6**a), and several studies were performed to identify the inherent factors contributing to the OER. Notably, among all the catalysts, CoPBO/Co_3_O_4_ exhibited the lowest overpotential, requiring just 270 mV to achieve a current density of 10 mA cm^−2^. In comparison, RuO_2_, Co‐MOF, MOF‐derived Co_3_O_4_, and CoPBO@Co‐MOF required overpotentials of 360, 410, 340, and 300 mV, respectively. Similar to the observations in the HER, the presence of CoPBO over Co‐MOF is beneficial for OER, as depicted by comparing with the control samples of CoBO@Co‐MOF (440 mV) and CoPO@Co‐MOF (440 mV) (Figure S19a, Supporting Information). Nevertheless, the overpotential value for CoPBO@Co‐MOF (300 mV) closely resembled that of pristine CoPBO powder (290 mV) and CoPBO@Co_3_O_4_ (300 mV) (Figure S20a, Supporting Information), where CoPBO is present on the surface of all the catalyst. Moreover, the purpose of integrating P and B was justified as higher overpotentials of 300 and 310 mV were exhibited by CoBO/Co_3_O_4_ and CoPO/Co_3_O_4_, respectively, that were fabricated using a similar technique as CoPBO/Co_3_O_4_ (Figure S21a, Supporting Information). These findings strongly suggest that the coexistence of a mixed phase of CoPBO and Co_3_O_4_ is highly favorable for not only HER but also for OER to establish the bifunctional nature of CoPBO/Co_3_O_4_ with remarkable activity for both half‐reactions. Comparing the overpotential values of recently reported electrocatalysts reveals that CoPBO/Co_3_O_4_ is among the most promising candidates for OER (Table S4, Supporting Information). The charge transfer resistance at the electrode/electrolyte interface is also minimal for CoPBO/Co_3_O_4_ (3.4 Ω), as substantiated by the Nyquist plots (Figure 6b and S19b, S20b, S21b, Supporting Information). Furthermore, the Tafel slope (Figure 6c and S19c, S20c, S21c, Supporting Information) for CoPBO/Co_3_O_4_ (61 mV dec^−1^) is the lowest, indicating fast reaction kinetics during the OER. The intrinsic activity per catalytic active site is remarkably higher for CoPBO/Co_3_O_4_, as depicted by the TOF value (at *η* = 300 mV) of 0.010 atom^−1 ^s^−1^, which significantly supersedes the Co‐MOF (0.0000372 atom^−1 ^s^−1^), MOF‐derived Co_3_O_4_ (0.00194 atom^−1 ^s^−1^), and CoPBO@Co‐MOF (0.0029 atom^−1 ^s^−1^). Moreover, the ECSA and BET normalized LSVs (Figure S22, Supporting Information) further corroborate the intrinsically active nature of the surface of CoPBO/Co_3_O_4_ composite. To ensure that Pt contamination does not affect the OER activity, the LSVs were repeated using graphite as the counter electrode (Figure S23, Supporting Information)

**Figure 7:**
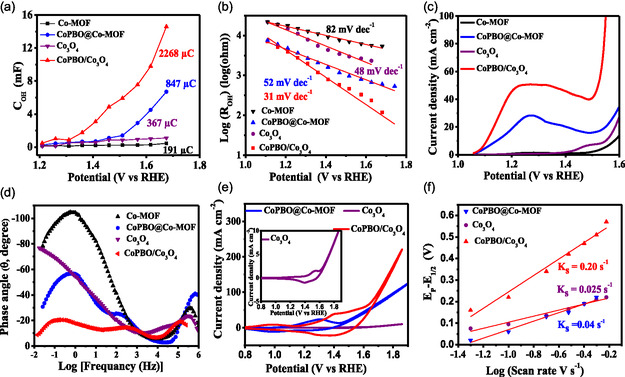
a) Plot of C_OH_ versus applied potential, b) EIS‐derived Tafel plots, c) LSV in the pre‐OER potential region acquired at a scan rate of 2 mV s^−1^, d) Bode plot acquired at 1.5 V (vs RHE), e) cyclic voltammetry at a scan rate of 50 mV s^−1^ with inset showing magnified CV plot of Co_3_O_4_, and f) plot of the difference between the redox peak of Co^3+^/Co^4+^ versus the log of scan rate for OER of Co‐MOF, CoPBO@Co‐MOF, MOF‐derived Co_3_O_4_, and CoPBO/Co_3_O_4_ recorded in 1 M KOH.

In alkaline electrolytes, the conventional OER mechanism over TMO relies on the adsorption of OH^−^ ions and the subsequent desorption of O_2_ molecules through deprotonation over catalytically active metal sites. These steps involve the formation of intermediates (M–O, M–OH, M–OOH), which are recognized as the rate‐determining steps (RDS) for OER and were identified using various methods. To track the adsorption of OH^−^ ions, operando EIS measurement was performed at different potential values (Figure S24, Supporting Information). The recorded Nyquist plots with three semicircles were fitted with three parallel circuits of resistance and capacitance (Figure S25, Supporting Information).^[^ ^28^ ^]^ The first parallel RC circuit corresponds to the surface charge transfer resistance (*R*_CT_) at the electrode/electrolyte interface and double‐layer surface capacitance (*C*_dl_). The adsorption behavior of the reactant (OH^−^) on the catalyst surface is described by the second circuit, where *R*_OH_ and *C*_OH_ represent the resistance and pseudocapacitance for the adsorption of OH^−^ ions, respectively. The final *R*_f_ *C*_f_ circuit corresponds to the resistance and dielectric properties offered by the underlying compact catalyst film. All the resistance and capacitance values obtained after fitting with the circuits are summarized in Table S5, Supporting Information. The *R*_OH_ value decreases while *C*_OH_ increases as the potential rises, with maximum change noticed for CoPBO/Co_3_O_4_ catalyst. The plot of *C*_OH_ versus potential (**Figure** **7**a and S26a, Supporting Information) was integrated to quantify the OH^−^ adsorption charge (*Q*_OH_) on the surface of the electrocatalyst during OER. With the highest *Q*_H_ value of 2268 μC, the CoPBO/Co_3_O_4_ composite showed the most improved OH^−^ adsorption followed by CoPBO@Co‐MOF (847 μC), MOF‐derived Co_3_O_4_ (367 μC), and Co‐MOF (191 μC). The Tafel slope values are determined by linear fitting the plots of log (*R*_OH_) versus *E* (Figure 7b and S26b, Supporting Information). The lowest Tafel slope value noticed for CoPBO/Co_3_O_4_ (31 mV dec^−1^) showcases the optimal condition produced on the surface for OH^−^ adsorption.

Although the OH^−^ is found to be conveniently adsorbed on the surface, it must be able to bind with metal to form the reactions intermediate (OH* and OOH*), which were investigated by examining the preoxidation peak formation. The formation of hydroxide/oxyhydroxide intermediates was probed by measuring LSV at a slow scan rate of 2 mV s^−1^ in the first OER cycle (Figure 7c). Both the electrocatalysts containing CoPBO show a peak at the lower potential region of 1.22–1.25 V corresponding to Co^2+^ to Co^3+^ conversion, indicative of the formation of CoOOH on the surface (Figure S27, Supporting Information).^[^ ^26^, ^29^ ^]^ A second peak at 1.38–1.39 V is linked with the conversion of Co^3+^ to Co^4+^ (CoO_2_), followed by the OER.^[^ ^28^, ^54^ ^]^ The reconstruction of the surface is also observed in the catalysts containing CoPBO, such as pristine CoPBO and CoPBO@Co_3_O_4_ (Figure S28, Supporting Information), which is consistent with the “precatalyst” theory asserting that metal borates and phosphides act as precatalysts to assist the formation of active oxyhydroxide species.^[^ ^1^, ^30^, ^47^ ^]^ This finding suggests that the CoPBO on the surface facilitates the reconstruction of the surface to generate CoOOH active species. The intensity of the preoxidation peak determines the degree of oxidation. Upon comparing CoPBO/Co_3_O_4_ with other counterparts containing CoPBO, the former produced the highest peak intensity with an elevated degree of oxidation (Figure 7c and S28, Supporting Information). This indicates a larger number of Co‐sites transform into CoOOH active species, thereby expediting the OER process. MOF‐derived Co_3_O_4_ only showed the formation of CoO_2_ at higher potential, but in Co‐MOF, neither of these peaks is visible. As the formation of CoOOH on the surface is a nonreversible process, XPS (Figure S29, Supporting Information) and Raman spectroscopy (Figure S30, Supporting Information) were used to investigate the surface of CoPBO/Co_3_O_4_ post‐OER (after 12 h of the chronoamperometric test). The metallic peak of Co completely disappears while the peak corresponding to Co^3+^ is shifted positively by 0.5–780.6 eV, which is attributed to the formation of CoOOH^[^ ^47^ ^]^ (Figure S29a, Supporting Information). The peak at 782.2 eV of Co^2+^ in Co_3_O_4_ is negatively shifted to 781.6 eV assigned to Co^2+^ in Co(OH)_2_. The intensity of the Co^2+^ peak is also noticed to diminish with the increase in Co^3+^ peak intensity, thus confirming the formation of CoOOH species. The Raman peak also validates the surface reconstruction of CoPBO/Co_3_O_4_ during OER by the evolution of peaks at 604 and 497 cm^−1^, designated to CoOOH,^[^ ^55^, ^56^ ^]^ from Co_3_O_4_ peaks of the pre‐OER electrocatalyst (Figure S30, Supporting Information). Moreover, the surface transformation was also clearly noticed in the morphological analysis (Figure S31, Supporting Information), where nanoparticles are converted into hexagonal sheet‐like morphology. These hexagonal structures are reflective indications of the formation of CoOOH, as reported by Chunduri et al.^[^ ^47^ ^]^ All these techniques provided tangible proof for the formation of CoOOH on the surface of CoPBO/Co_3_O_4_ in agreement with the transition metal phospho‐borate catalyst.^[^ ^47^ ^]^ XRD analysis (Figure S32, Supporting Information) reveals no detectable changes in the bulk structure of the catalyst despite significant surface reconstruction.

After scrutinizing the OH^−^ adsorption and formation of M‐OOH/OH, it is crucial to explore the deprotonation step of CoOOH, which further leads to O_2_ desorption. The deprotonation step was investigated by recording the Bode plots^[^ ^28^ ^]^ at different potentials (Figure S33, Supporting Information), and the plot recorded at 1.5 V is presented in Figure 7d and S26c, Supporting Information. The phase peaks at higher (10^2^–10^4^ Hz) and middle (10^0^–10^2^ Hz) frequency ranges were obtained by the surface oxidized species of Co–O_6_ and Co–O_4_,^[^ ^28^, ^57^, ^58^ ^]^ respectively. On the other hand, the phase peak at the lowest frequency is attributed to the deprotonation of Co–OOH. The first two phase peaks have approximately similar phase angles for all four electrocatalysts. Nevertheless, the phase peak for deprotonation is observed at a considerably lower phase angle for CoPBO/Co_3_O_4_ electrocatalyst compared to others, signifying faster deprotonation of CoOOH in CoPBO/Co_3_O_4_. Finally, the O_2_ desorption can be qualitatively evaluated by examining the preoxidation behavior of Co species. This was done by measuring the CV curves at different scan rates (Figure S34, Supporting Information) and investigating the evolution of the product from the surface on the basis of the Laviron equation.^[^ ^28^ ^]^ CV measured at a scan rate of 50 mV s^−1^ displayed the preoxidation peaks for Co^2+^/Co^3+^ and Co^3+^/Co^4+^ in CoPBO‐based electrocatalyst (Figure 7e and S26d, Supporting Information), while only a higher oxidation state peak is visible in MOF‐derived Co_3_O_4_ (inset of Figure 7e), and no clear peak was observed for Co‐MOF. These results are consistent with LSVs measured at slow scan rates (Figure 7c). The reduction peak of Co^4+^ to Co^3+^ in the cathodic region suggests a reversible reaction, while the absence of corresponding Co^3+^ to Co^2+^ cathodic peak indicates that the catalyst surface has undergone an irreversible surface reconstruction to form active CoOOH species for subsequent OER. The redox constant (*K*_s_) was determined by linearly fitting the plot of the difference between the redox peak of Co^3+^/Co^4+^ versus the log of scan rate (Figure 7f and S26e, Supporting Information). This *K*_s_ value depicts the formation of the product on the surface, which is the desorption of O_2_ in this case. The *K*_s_ value for CoPBO/Co_3_O_4_ (0.20 s^−1^) is considerably higher than that obtained for CoPBO@Co‐MOF (0.04 s^−1^) and MOF‐derived Co_3_O_4_ (0.025 s^−1^), suggesting accelerated O_2_ desorption over the surface of CoPBO/Co_3_O_4_.^[^ ^28^ ^]^ All the above findings obtained through kinetic studies infer that CoPBO/Co_3_O_4_ renders an optimal surface to aid the adsorption of OH^−^ ions with superior charge transfer and faster deprotonation to improve the O_2_ desorption. This is attributed to the mixed phase of crystalline Co_3_O_4_ and amorphous CoPBO in the optimized CoPBO/Co_3_O_4_ composite as pristine CoPBO and CoPBO@Co_3_O_4_ showed lower OER kinetics (Figure S26, Supporting Information).

**Scheme 1:**
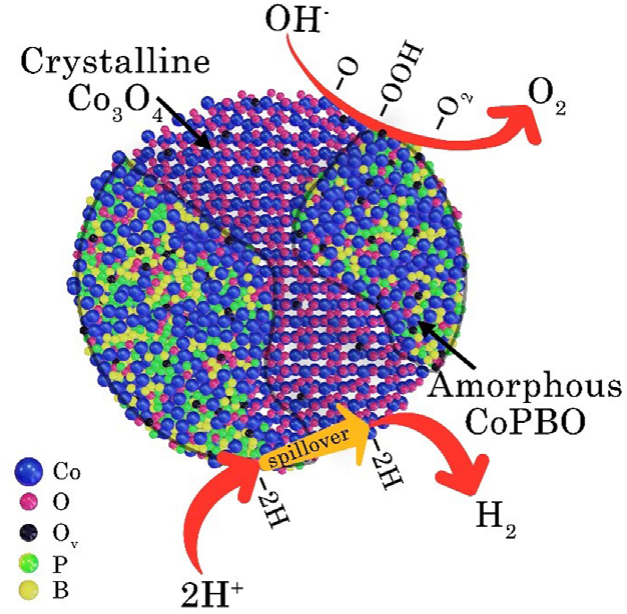
Schematic diagram to show the bifunctional character of CoPBO/Co_3_O_4_ composite.

Previous studies have established that oxygen vacancies within TMO function as mediators, expediting the adsorption of OH^−^ ions by altering the surface electronic structure and amplifying the overall OER activity. As established by XPS, after the phospho‐boronization process, the amount of O_v_ increases on the surface, with the highest amount seen in the mixed phase CoPBO/Co_3_O_4_ composite. Thus, the overwhelming OER activity in CoPBO/Co_3_O_4_ is, in fact, credited to the abundance of surface oxygen vacancies. The metal sites surrounded with O_v_ (M_ov_) readily interact with the oxygen from OH^−^ ions to form intermediates due to unfilled antibonding states of M_ov_. The bonding strength of these intermediates is decided by the difference between the highest occupied *d*‐state (denoted as *E*_d_) and the Fermi level (*E*_f_). The presence of O_v_ places the *E*_d_ at higher levels, thus increasing the gap between *E*_f_ and *E*_d_, which leads to the stronger binding of the intermediates to the surface which was confirmed by Xiao et al.^[^ ^28^ ^]^ by DFT‐based calculations and experimental measurements, where the RDS for O_v_‐rich Co_3_O_4_ was the conversion of OOH* to O_2_ rather than the formation of OOH* as in the case of Co_3_O_4_. This finding was attributed to the enhanced binding strength of O* and OOH* intermediates with Co‐sites surrounded by O_v_. Likewise, in the present case, the CoPBO/Co_3_O_4_ catalyst is rich with M_ov_ and provides a suitable surface for OH^−^ adsorption, which binds with optimal bonding strength. The existence of O_v_ has also been demonstrated to enhance the electrical conductivity of metal oxide and accelerate charge transfer, thereby increasing the OER activity. Particularly in Co_3_O_4_, these oxygen vacancies significantly reduce the bandgap, enhancing the conductivity. Notably, the present configuration of CoPBO/Co_3_O_4_, characterized by the highest concentration of O_v_, exhibits the lowest *R*_CT_ and *R*_f_, consequently contributing to its superior OER performance. All the above findings depict that the amorphous CoPBO and crystalline Co_3_O_4_ composite offer unique surfaces to improvise both HER and OER kinetics through the HS mechanism and O_v_ formation, respectively, to tender bifunctional characteristics as depicted in **Scheme** **1**. The crystalline phase maintains structural stability, while the amorphous phase offers unsaturated undercoordinated sites to enhance the adsorption of the reactants, hence offering a large number of active sites.^[^ ^59^ ^]^ Moreover, the combination of ordered and disordered atomic arrangement formulates the specific reaction steps to accelerate the reaction kinetics on the surface. The metastable and flexible structure of the amorphous phase allows self‐reconstruction under the reaction environment, as observed during OER.^[^ ^59^ ^]^

**Figure 8:**
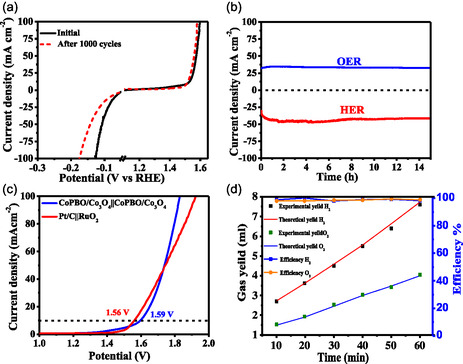
a) Linear polarization curves of CoPBO/Co_3_O_4_ before and after 1000 cycles for OER and HER, b) chronoamperometric test of CoPBO/Co_3_O_4_ for OER (1.53 V vs RHE) and HER (−0.07 V vs RHE), c) LSV for two‐electrode setup using CoPBO/Co_3_O_4_||CoPBO/Co_3_O_4_, and d) Faradaic efficiency plot of CoPBO/Co_3_O_4_ recorded in 1 M KOH.

Upon comprehending the intrinsic qualities of CoPBO/Co_3_O_4_ as an exceptional bifunctional catalyst, it becomes crucial to assess its commercial viability. To evaluate the long‐term durability of the CoPBO/Co_3_O_4_ for HER and OER, extensive testing was conducted, which included subjecting them to 1000 cycles of cathodic and anodic cycles and 15 h of long‐term chronoamperometric test. Notably, CoPBO/Co_3_O_4_ exhibits no loss in OER activity, but a marginal decline in HER activity can be due to the agglomeration of particles after 1000 cycles, highlighting its endurance for OER and HER (**Figure** **8**a). During the chronoamperometric test, CoPBO/Co_3_O_4_ could maintain the constant current density over the entire 15 h test duration, showcasing its long‐term stability (Figure 8b). The CoPBO/Co_3_O_4_ was used in two electrode setups, where CoPBO/Co_3_O_4_ on glassy carbon electrode (GCE) was used as the catalyst for both the cathode and anode. The overall cell voltage of 1.59 V (without iR correction) is required to reach 10 mA cm^−2^ by CoPBO/Co_3_O_4_||CoPBO/Co_3_O_4_, which is comparable (≈30 mV more) to that achieved by using Pt/C||RuO_2_ in a two‐electrode assembly (Figure 8c). At 100 mA cm^−2^, the present non‐noble catalyst requires merely 1.82 V that surpass the noble metal catalyst, which needs 1.92 V. Upon comparison with reported transition metal electrocatalysts tested on planar GCEs (Table S6, Supporting Information), the present catalyst stands out in overall water splitting. To quantify H_2_ and O_2_ evolution reactions and establish that the produced current is due to the evolved gases, the volume of the gases was measured during the two‐electrode measurements (for CoPBO/Co_3_O_4_@NF||CoPBO/Co_3_O_4_@NF) and matched with the theoretically expected volumes, yielding a Faradaic efficiency of ≈100% (Figure 8d). Furthermore, the same two‐electrode system employing the CoPBO/Co_3_O_4_@NF catalyst was operated using a single AA battery that provides 1.5 V. As visible in the video (Video S1, Supporting Information), the bubble formation was clearly spotted on both electrodes even at this minimum applied voltage. The above findings reveal that the CoPBO/Co_3_O_4_ composite not only has all the traits for effective overall water splitting but can also blend in the commercial setup.

## 3 Conclusion

In summary, the optimized CoPBO/Co_3_O_4_ composite catalyst possessing a mixed amorphous–crystalline phase turns out to be an ideal bifunctional catalyst for alkaline water electrolysis. The superior HER performance is driven by the HS mechanism that occurs due to the complementing H‐adsorption Gibbs free energy of CoPBO and Co_3_O_4_ and a small difference in their work functions. Likewise, the abundant number of surface oxygen vacancies produced due to the phospho‐boronization process assists in OH^−^ adsorption and drives the OER kinetics more efficiently. The composite catalyst exhibits considerable robustness, as established by the stability and recycling tests. In two‐electrode assemblies, they outclass the state‐of‐the‐art PGM catalysts at higher current densities, further establishing their performance. This work exemplifies how non‐noble electrocatalyst composites can be tailored to achieve bifunctionality and, consequently, new breakthroughs in alkaline water electrolysis.

## 4 Experimental Section

4.1

4.1.1

##### Synthesis of Co‐MOF

Co‐MOF was synthesized using a solvothermal method. In brief, 0.989 g of cobalt nitrate (Co (NO_3_)_2_.6H_2_O) and 0.168 g of terephthalic acid (H_2_BDC) were dissolved into 25.6 mL of *N*,*N*‐dimethylformamide (DMF) under constant stirring for 10 mins at room temperature. This solution mixture was transferred into a 70 mL Teflon‐lined stainless‐steel autoclave and kept in the oven at an optimized temperature and time of 120 °C and 20 h, respectively. The obtained product was centrifuged and washed with DMF 3 times, followed by ethanol. The final precipitate was dried in an oven at 60 °C for 12 h to obtain Co‐MOF powder.^[^ ^60^ ^]^

##### Synthesis of CoPBO on Co‐MOF (CoPBO@Co‐MOF)

CoPBO@Co‐MOF was synthesized by using a chemical reduction method where sodium borohydride (NaBH_4_) and sodium hypophosphite (NaH_2_PO_2_) were used as boron and phosphorous sources, respectively, as well as reducing agents. 20 mg of as‐prepared Co‐MOF was dispersed into 12 mL of deionized (DI) water under constant stirring for 30 min at room temperature. Later, NaH_2_PO_2_ (0.79 mmol) and NaBH_4_ (0.79 mmol) with equal molar ratios were added to the Co‐MOF solution under magnetic stirring at room temperature. The pink colour Co‐MOF turned black immediately after the addition of the reducing agents with noticeable effervescence. Once the effervescence ceased, after 1 h, the black precipitate was separated through centrifugation and washed 3–4 times with DI water and ethanol. The total weight ratio of Co‐MOF to (B + P) was maintained at 1:3 with a B to P molar ratio of 1. These optimal ratios were selected based on electrochemical performance recorded for the electrocatalyst with different ratios. Similarly, CoBO@Co‐MOF and CoPO@Co‐MOF were fabricated in the absence of NaH_2_PO_2_ and NaBH_4_, respectively, and the weight ratio of Co‐MOF to B or P was maintained at 1:3.

##### Synthesis of CoPBO and Co_3_O_4_ Composite (CoPBO/Co_3_O_4_)

As‐synthesized Co‐MOF was pyrolyzed in air at 700 °C for 2 h to form MOF‐derived Co_3_O_4_. Again, here the temperature range was varied from 350 to 800 °C, and 700 °C was found to be optimum for pyrolysis. 40 mg of this Co_3_O_4_ powder was dispersed into 50 mL of DI water under constant stirring for 30 min at room temperature. To incorporate P and B, a similar method of chemical reduction was adopted by using NaH_2_PO_2_ (31.72 mmol) and NaBH_4_ (31.72 mmol), respectively. However, in this case, due to the stable nature of Co_3_O_4_, a higher amount of reducing agent was used as compared to that of Co‐MOF with a weight ratio of Co to (B + P) as 1:60 while maintaining the B to P molar ratio to 1. Similarly, CoBO/Co_3_O_4_ and CoPO/Co_3_O_4_ were synthesized in the absence of NaH_2_PO_2_ and NaBH_4_, respectively.

##### Synthesis of CoPBO@Co_3_O_4_ and CoPBO

For comparison with CoPBO/Co_3_O_4_, Co oxide NPs were also synthesized using a conventional coprecipitation method as reported in previous work.^[^ ^54^ ^]^ The prepared nanoparticles were pyrolyzed in air at 700 °C for 2 h to form Co_3_O_4_. The surface phospho‐boronization was achieved using a similar method of chemical reduction, as stated in previous Section 4.3, to form a CoPBO@Co_3_O_4_ electrocatalyst. Similarly, pristine CoPBO powder was synthesized by chemically reducing cobalt chloride with the two reducing agents, as also detailed in our previous work.^[^ ^30^ ^]^

##### Material Characterization

XRD patterns were obtained on a Rigaku (Mini flex) system equipped with a radiation source of Cu Kα (*λ* = 1.5441 nm). The Raman spectra of the catalysts were recorded using a Renishaw instrument with a 532 nm laser as an excitation source. SEM (JEOL 7001F) and TEM (JEOL, JEM 2100F model, FEG 200 kV) were used to investigate the morphology, while HRTEM and SAED patterns were utilized to study the structural properties at the nanoscale. The elemental distribution was analyzed using area mapping through an energy‐dispersive spectrometer during SEM imaging. XPS (Versaprobe III, PHI) was used to investigate the elemental composition and chemical state of the electrocatalyst surface. The calibration of binding energies for each element was achieved by using the C 1*s* spectrum as the reference. BET technique was used to determine the physical surface area of the powder electrocatalyst using the N_2_ adsorption method with the multipoint surface analyzer (Micrometric 3 flex 3500 gas adsorption analyzer). ESR spectra (JES–FA200) were measured to identify the presence of oxygen vacancies in the electrocatalyst.

##### Electrochemical Measurements

A 3 mm GCE coated with as‐prepared catalyst was used as the working electrode. For the catalyst deposition on GCE, a homogeneous ink was prepared by dispersing 5 mg of catalyst powder in 1 mL ethanol through sonication. A binder solution was prepared by adding 40 μL of Nafion into 1 mL of ethanol. Prior to the deposition of the 20 μL of catalyst ink, 10 μL of binder solution was drop‐casted first on GCE and dried under an IR lamp. The catalyst loading was maintained at 1.42 mg cm^−2^ for each electrochemical measurement. The electrochemical activity of the electrocatalyst was determined by using an electrochemical workstation (CH instrument‐CH 16011E) in a standard three‐electrode configuration. A Pt electrode (2 mm) and a saturated calomel electrode (SCE) were used as the counter and reference electrodes, respectively, with 1 M KOH (pH = 14) solution as the electrolyte. The electrolyte was continuously stirred at room temperature to prevent the accumulation of bubbles on the surface of the working and counter electrodes. To determine the HER and OER overpotentials, LSV curves were recorded in the potential range of −1.4 to −1.06 V (vs SCE) and 0–0.8V (vs SCE), respectively, at a scan rate of 10 mV s^−1^. All the measured potentials were converted with respect to the reversible hydrogen electrode (RHE) by using the following Nernst equation: *E*_RHE_ = *E*_SCE_ + 0.059 × pH (where *E*_SCE_ = +0.241 V and pH = 14). EIS was performed by giving an input sine wave of amplitude 10 mV in the frequency range of 1 MHz–0.01 Hz to determine the charge transfer resistance (*R*_CT_) and solution resistance (*R*_u_) from the Nyquist plots. The LSVs were iR‐corrected to remove the effect of solution resistance. The Tafel slope of each electrocatalyst was determined by fitting a plot of log (current density *I*_0_) versus overpotential (*η*) to study the reaction kinetics. ECSA was calculated by measuring the double‐layer capacitance (*C*_dl_) obtained from CV performed at different scan rates (20, 40, 60, 80, 100, and 120 mV s^−1^) in the non‐Faradaic range within ±100 mV across the open‐circuit potential (OCP). Based on the obtained CVs, the difference in peak current densities between the cathodic and anodic sides (Δ*J*) at the OCP is plotted against the corresponding scan rates to determine the value of *C*_dl_. The adsorption and desorption kinetic studies were conducted by measuring Nyquist plots at different potential values and CV curves at different scan rates. The value of TOF of electrocatalysts was calculated using the formula reported by Jinhui Tong et al.^[^ ^61^ ^]^ The overall water splitting was performed in a two‐electrode assembly using the best electrocatalyst (CoPBO/Co_3_O_4_) coated on GCE as both electrodes. The stability of the electrocatalyst was investigated by chronoamperometric measurement for 15 h and LSV measurements for 1000 cycles. Hoffman apparatus was utilized to assess the Faradaic efficiency for both HER and OER. For this measurement, the electrocatalyst was drop‐casted on pretreated Ni foam (0.5 × 1 cm) and used as both electrodes. Prior to conducting the measurements, a constant current for 30 min was applied to precondition the electrode surface and eliminate atmospheric gases from the apparatus. Subsequently, the volume of H_2_ and O_2_ gases produced was measured on each side of the electrodes and compared with the theoretically calculated values of H_2_ and O_2_ gas to determine the Faradaic efficiency.

## Conflict of Interest

The authors declare no conflict of interest.

## Author Contributions

The manuscript was written with contributions from all authors. All authors have given approval to the final version of the manuscript. **Rinkoo Bhabal**: Experimental measurement; Data analysis; Manuscript preparation. **Aniruddha Bhide:** Experimental measurement; Data analysis; Manuscript preparation. **Suraj Gupta:** Data analysis; Methodology; Writing; Reviewing manuscript, **Rohan Fernandes:** Experimental measurement; Data analysis; Manuscript preparation. **Nainesh Patel:** Supervision; Conceptualization; Finalizing manuscript; Funding acquisition.

## Supporting information

Supplementary Material

## Data Availability

The data that support the findings of this study are available from the corresponding author upon reasonable request.
