# Supplementary material for: Maximizing Bifunctionality for Overall Water Splitting by Integrating H2 Spillover and Oxygen Vacancies in CoPBO/Co3O4 Composite Catalyst
Source: Small Sci. 2024 Nov 10;4(12):2400343. doi: 10.1002/smsc.202400343 (PMC11935209; doi:10.1002/smsc.202400343)
Supplement: Supplementary file 1 — Supplementary Material [file SMSC-4-2400343-s001.zip › smsc202400343-sup-0001-SuppData-S1.pdf]

Maximizing Bifunctionality for Overall Water Splitting by IntegratingH_2 Spillover and Oxygen Vacancies in CoPBO/Co_3O_4 Composite Catalyst

Rinkoo Bhabal[1], Aniruddha Bhide[1], Suraj Gupta[2], Rohan Fernandes[1], Nainesh Patel[1*]*

[1]*Department of Physics and Electronics, Christ University, Bengaluru, 560029, India*

[2]*Advanced Materials Department, Jožef Stefan Institute, Jamova 39, 1000 Ljubljana, Slovenia*

***Corresponding author:**

Dr. Nainesh Patel, nainesh.patel@christuniversity.in

Dr. Suraj Gupta, suraj.gupta@ijs.si

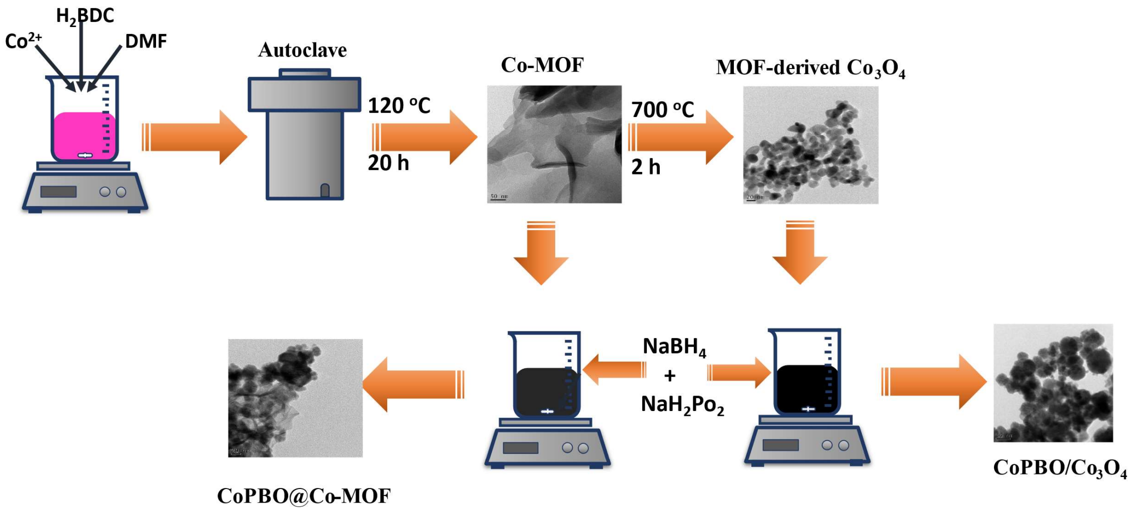

Scheme S1: Synthesis process of the electrocatalysts

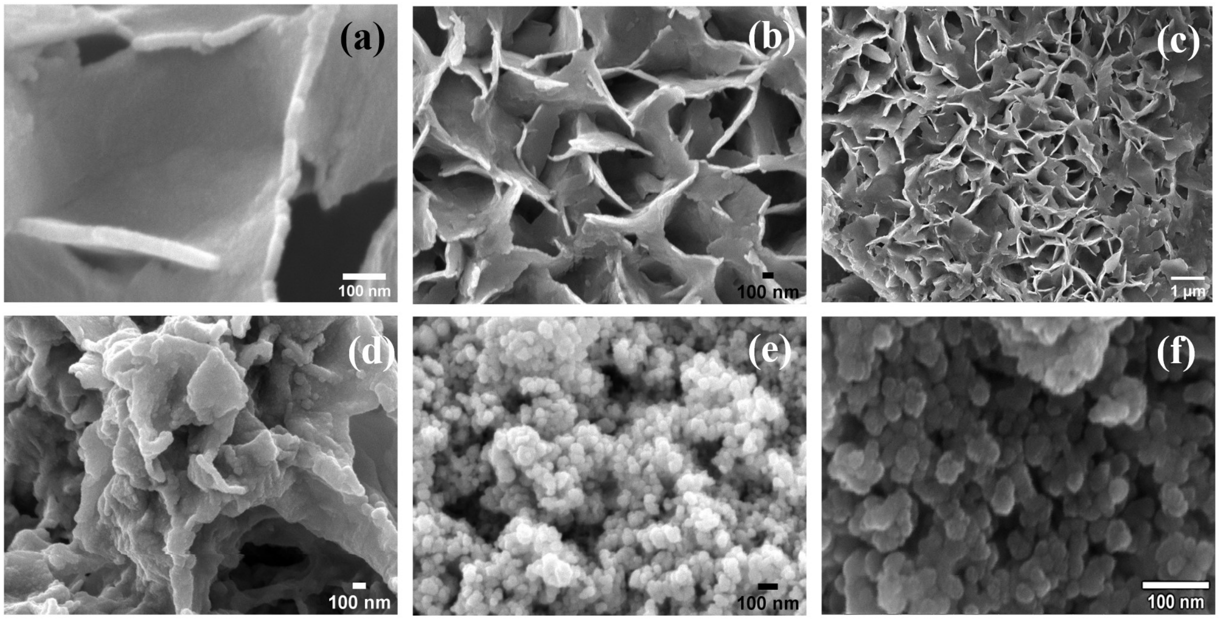

Figure S1: SEM images of (a-c) Co-MOF, (d) CoPBO@Co-MOF, (e) MOF-derived Co_3O_4, and (f) CoPBO/Co_3O_4 electrocatalyst.

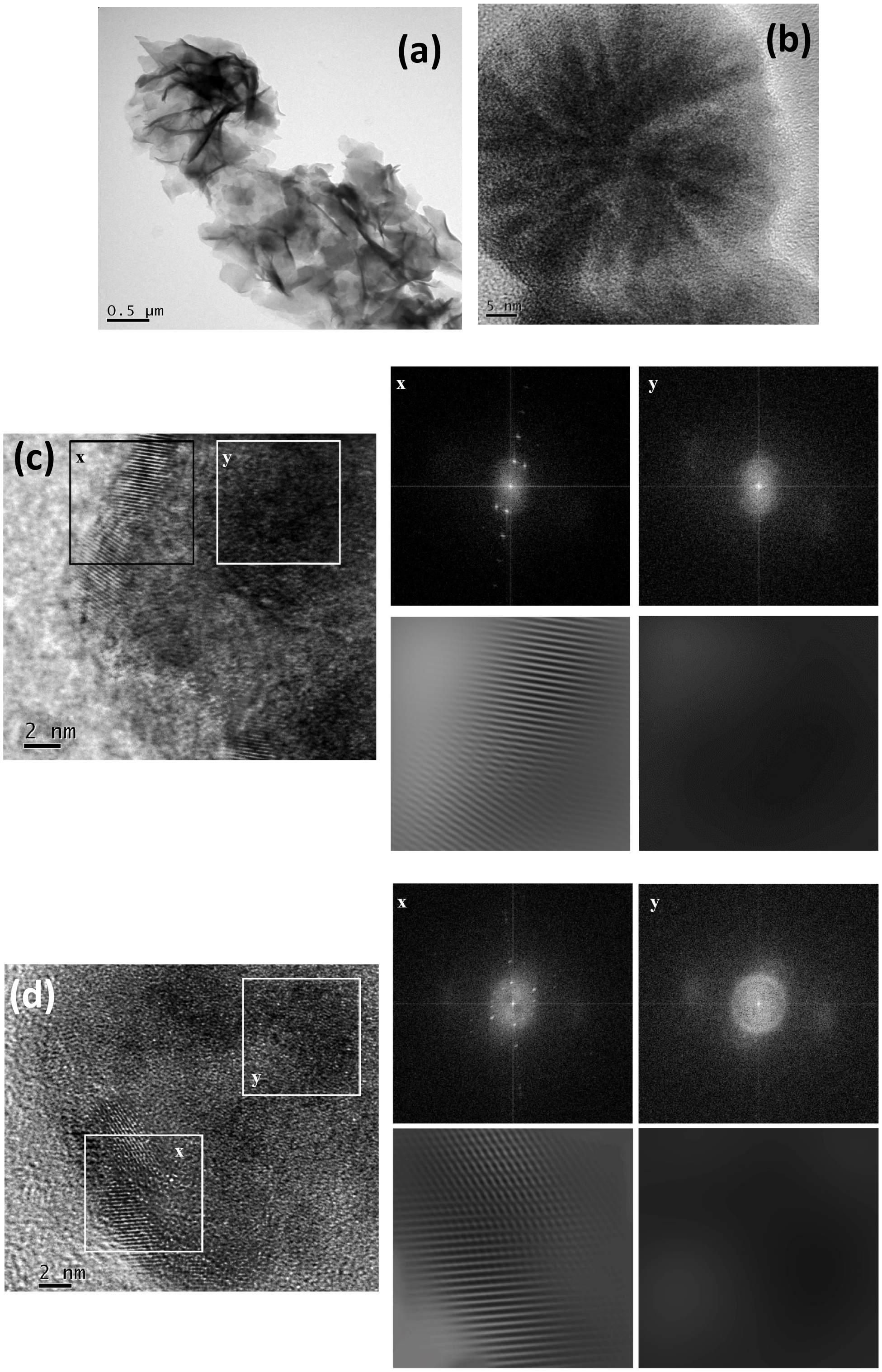

Figure S2: TEM images of (a) Co-MOF, (b) CoPBO/Co_3O_4, HRTEM images, and corresponding FFT and Inverse FFT image of (c) CoPBO@Co-MOF, and (d) CoPBO/Co_3O_4

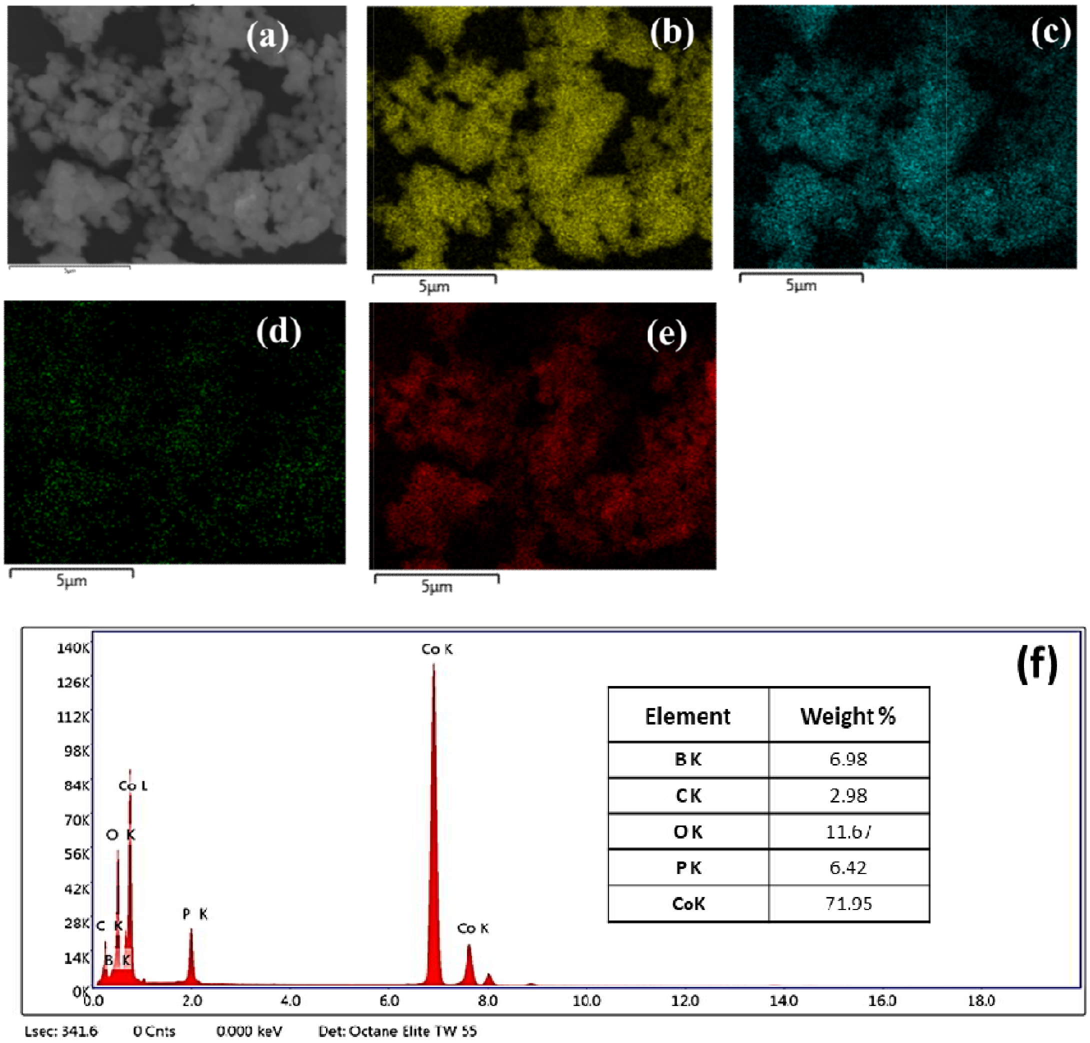

Figure S3:(a) Reference SEM image of CoPBO/Co_3O_4 and corresponding elemental mapping of (b) Co, (c)P, (d) B,(e)O,and (f) EDAX spectra obtained from TEM image.

Supplementary Table S1: The O_v/M-O peak ratios of CoPBO@Co-MOF, Co_3O_4, CoPBO/Co_3O_4

Electrocatalyst	Area under the peak		
	**M-O**	**O_v**	**O_v/M-O**
CoPBO@Co-MOF	17.84	51.7	2.89
Co_3O_4	7.35	60.39	0.29
CoPBO/Co_3O_4	40.24	59.76	8.21

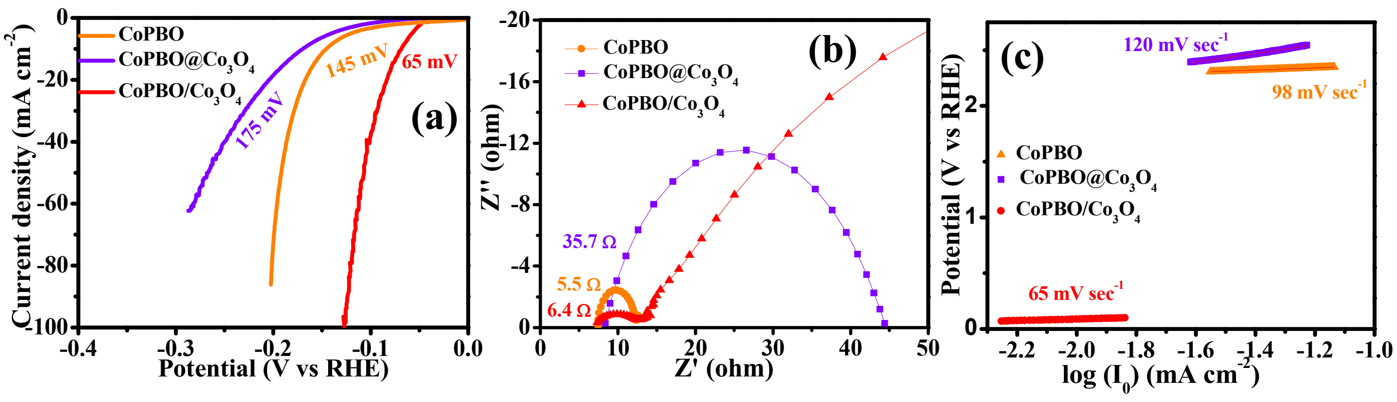

Figure S4:(a) Linear polarization curves, (b) Electrochemical Impedance Spectra, (c) Tafel plot of CoPBO, CoPBO@Co_3O_4, CoPBO/Co_3O_4forHER

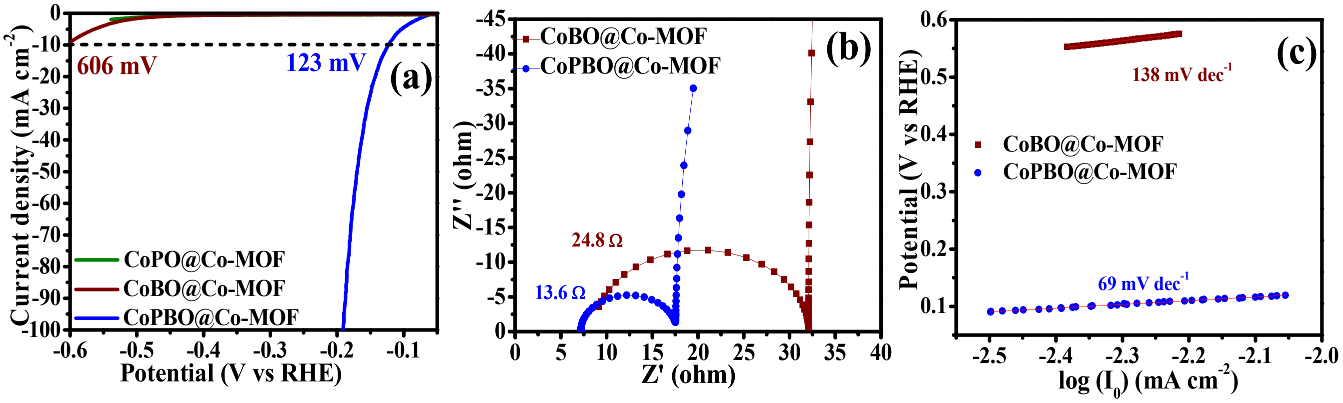

Figure S5:(a)Linear polarization curves, (b) Electrochemical impedance spectra, (c) Tafel plot of CoPO@Co-MOF,CoBO@Co-MOF, CoPBO@Co-MOF for HER recorded in 1M KOH.

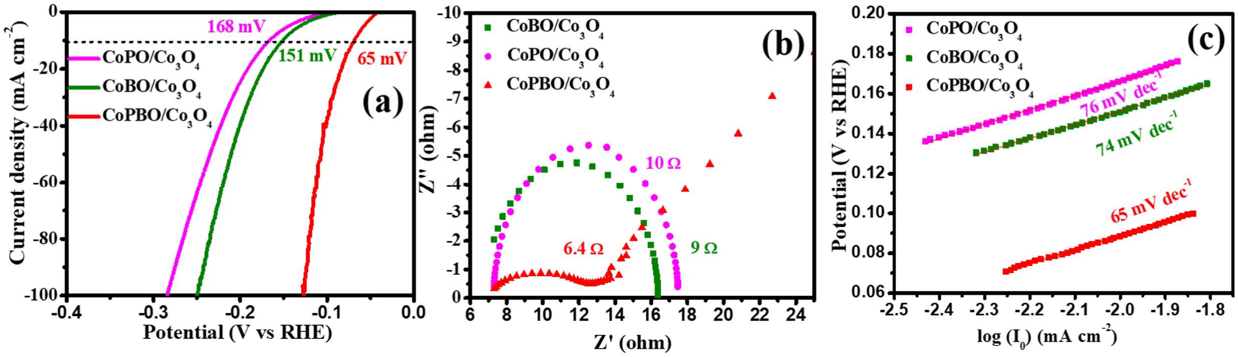

Figure S6: (a) Linear polarization curves, (b) Electrochemical impedance spectra, (c) Tafel plot of CoPO/Co_3O_4, CoBO/Co_3O_4, CoPBO/Co_3O_4 for HER recorded in 1M KOH.

Supplementary Table S2: Recently reported borides and phosphides-based electrocatalyst

Electrocatalyst	Overpotential	Ref. no.

performance for HER in alkaline medium.

	(mV@10mA cm^{-2})	
CoPB	145	[1]
CoWPB	72	[2]
CoWB	97	[3]
WB_2	198	[4]
Co_1-Fe_1-B-P	173	[5]
Ni-P film/Cu foil	93	[6]
Ni_2P/rGO NCs	142	[7]
NiFeP	182	[8]
NiCoFeP	56	[9]
Ni_xP/CNT	90	[10]
Ni_2P@PCG	150	[11]
NiSP	68	[12]
Ni_2P/C@NF	97	[13]
$Co_{0.6}Mo_{1.4}N_2$	200	[14]
NiB-400	144	[15]
$Fe(PO_3)_2$@Cu_3P	108	[16]
**CoPBO/Co_3O_4**	**65**	**This work**

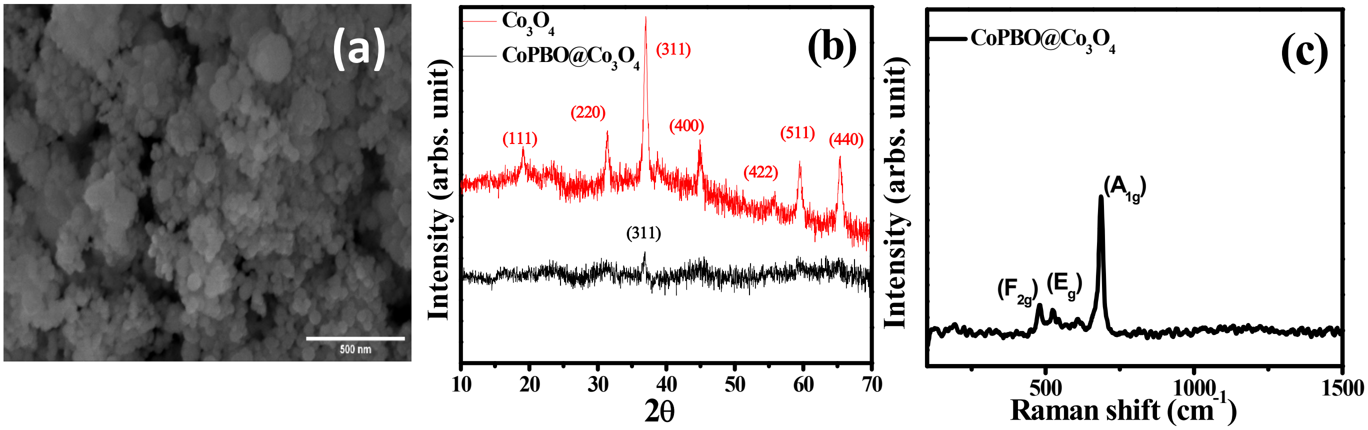

Figure S7:(a) SEM image, (b) XRD pattern, (c) Raman spectra of CoPBO@Co_3O_4

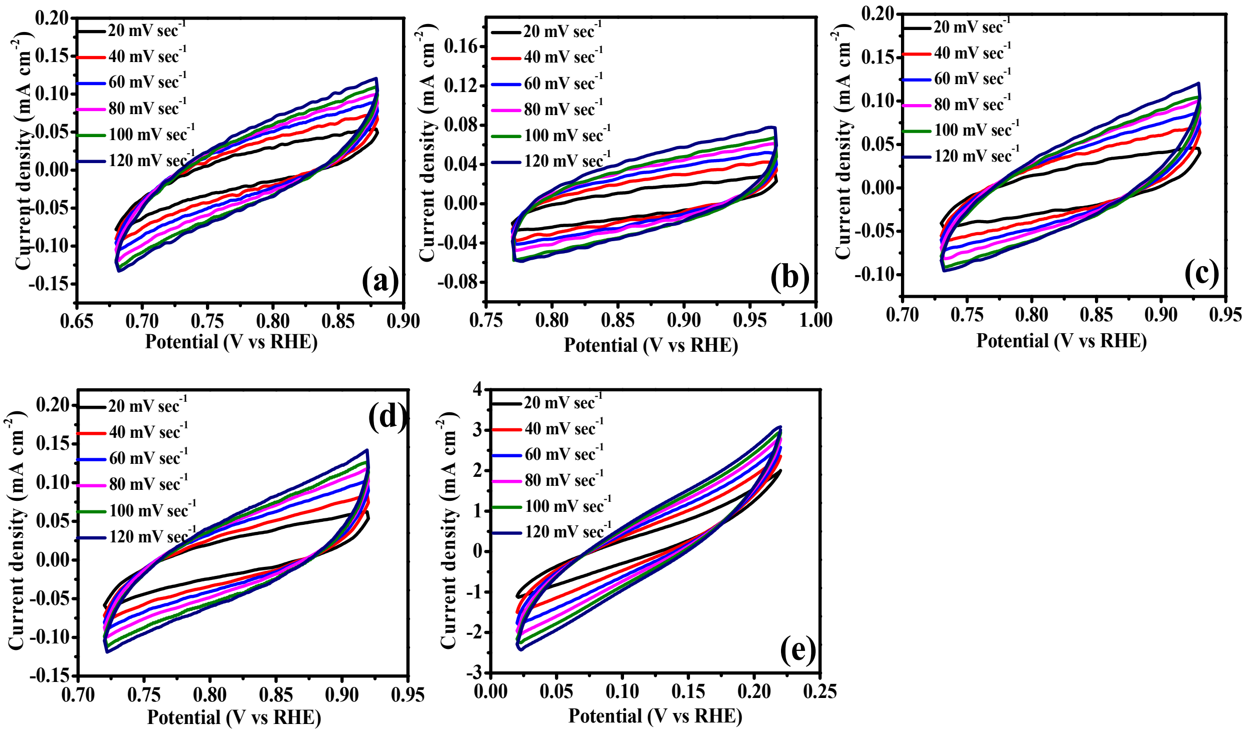

Figure S8: Cyclic voltammetry of (a) Co-MOF, (b) CoBO@Co-MOF, (c) CoPBO@Co-MOF, (d)MOF-derived Co_3O_4, and (e)CoPBO/Co_3O_4

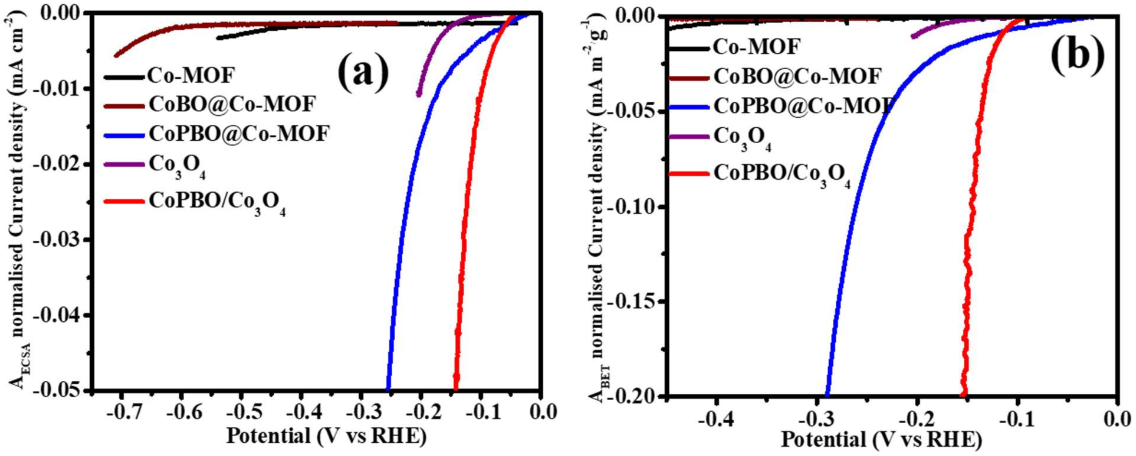

Figure S9: Electrochemical surface area (a) and BET surface area (b) normalized linear sweep voltammograms for HER of Co-MOF, CoBO@Co-MOF, CoPBO@Co-MOF, MOF-derived Co_3O_4, and CoPBO/Co_3O_4 for HER

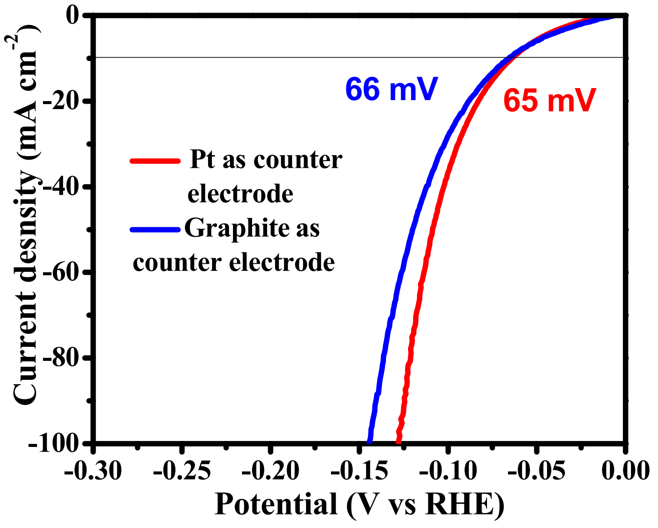

Figure S10:Linear polarization curve of CoPBO/$Co_3O_4$with Pt and graphite as counter for HER

Methodology to calculate work function:

Density functional theory calculations were carried out using the code Quantum Espresso with plane wave basis and PBE-GGA exchange correlation functional. The k-mesh grid was set to 6×6×1 for the accurate Brillouin zone integration. The planewave cut-off was kept to be 60 Ry to properly expand the and represent the wavefunctions. In order to calculate the workfunction of a $CoB_{0.5}P_{0.5}$ compound, a (001) slab of thickness 4.83 Å was used with a sufficient vacuum of 45 Å along the z direction. For the 50 % of P at the B sites, an 8-atom unit cell was used as shown in figure below. The table shows lattice parameters and the work function of the $CoB_{0.5}P_{0.5}$. The phosphorus atoms reside at the topmost and bottommost atomic layers. The workfunction () of this slab was calculated using the equation. 1

…(1)

Compound	Lattice parameters		Workfunction (eV)
	a (Å)	b (Å)	
$CoB_{0.5}P_{0.5}$	3.05	3.86	5.27

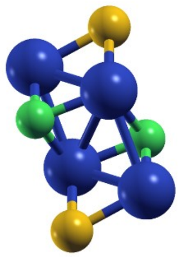

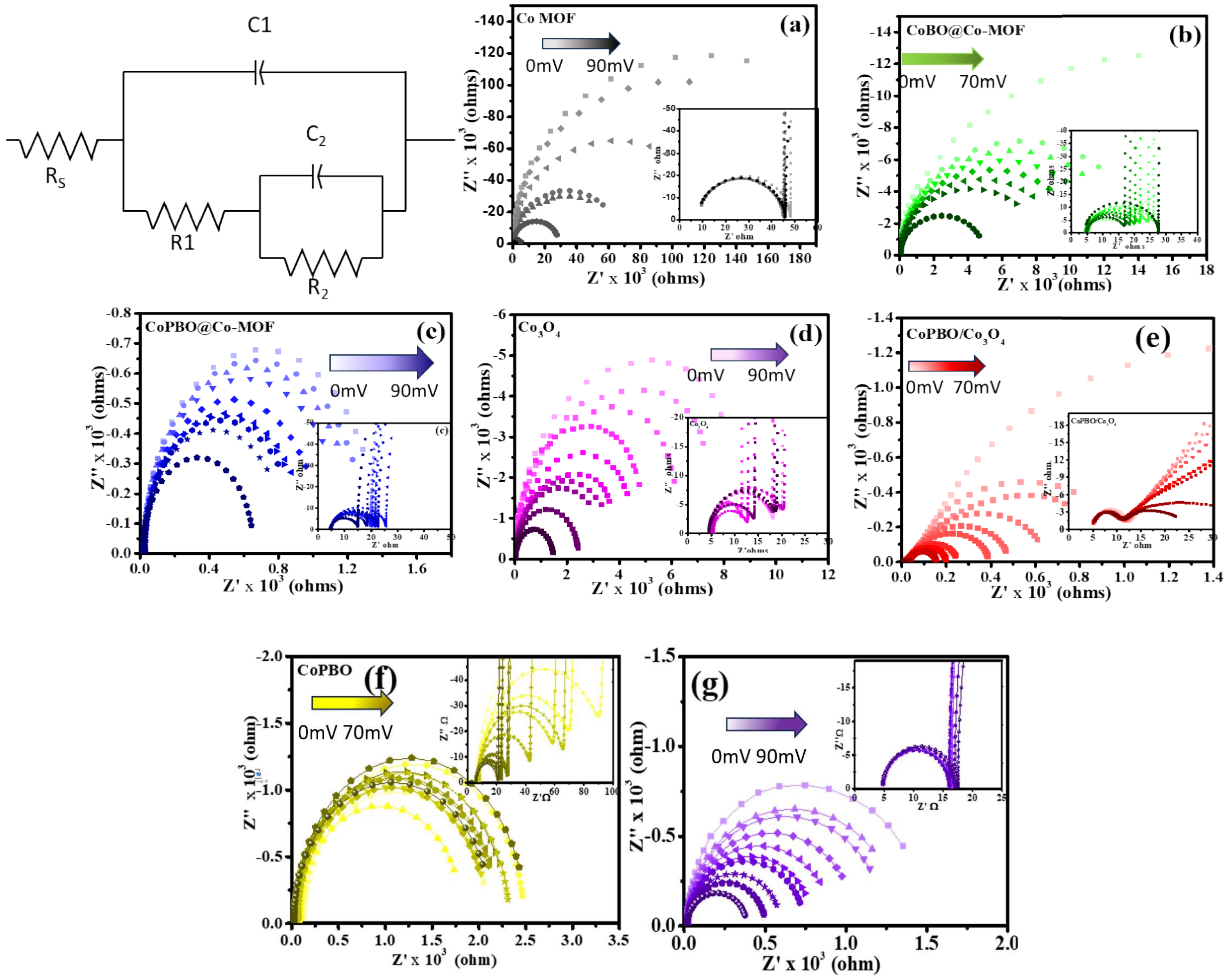

Figure S11: Electrochemical impedance spectra of (a) Co-MOF, (b) CoBO@Co-MOF, (c) CoPBO@Co-MOF, (d) MOF-derived Co_3O_4, (e) CoPBO/Co_3O_4, (f) CoPBO, and (g) CoPBO@Co_3O_4 at different potentials for HER in 1M KOH

Supplementary Table S3: The fitted parameters of the EIS data of Co-MOF, CoPBO@Co-MOF, MOF-derived Co_3O_4, CoPBO/Co_3O_4, CoPBO, and CoPBO@Co_3O_4 for HER.

Electrocatalyst	η mV	Rs (Ω)	R_1 (Ω)	R_2 (Ω)	C_2 (F)
Co-MOF	0	4.6	39	14656	0.00043
	10	4.8	37.1	110562	0.00049
	20	4.8	37	95932	0.00052
	30	4.9	36.6	56432	0.00055
	40	4.8	37.1	47712	0.00061
	50	5.1	37.3	27012	0.00067
	60	4.8	37.3	5173	0.00073
	70	5.2	37.9	1116	0.00078
CoPBO@Co-MOF	0	4.9	15.9	1277.8	0.00056
	10	4.8	15	1218.8	0.00061
	20	4.8	15	1228.8	0.00068
	30	4.8	16.6	1043.8	0.00074
	40	4.8	15.4	947.9	0.00083
	50	4.9	16.5	864	0.0010
	60	4.8	16.5	821	0.0011
	70	4.9	17.7	787.1	0.0012
	80	4.8	13.5	724.3	0.0013
	90	4.8	10.14	633.7	0.0016
MOF-dervied Co_3O_4	0	4.9	14.9	7877	0.000006
	10	4.8	14.7	7295	0.00003
	20	4.8	14.7	61086	0.00002
	30	5.2	14.6	4754	0.00006
	40	5.1	14.2	4384	0.00010
	50	4.9	14.5	3694	0.00012
	60	4.8	14.2	3396	0.00013
	70	4.8	14.9	2809	0.00015
	80	4.8	14.4	2403	0.00025
	90	4.9	14.3	1445	0.00027
CoPBO/Co_3O_4	0	5	6.4	1370.6	0.001
	10	5	6.4	767	0.0012
	20	4.9	6.4	726.3	0.0013
	30	5.1	6.3	599.7	0.0014
	40	4.9	6.4	460.59	0.0017
	50	5	6.4	230.7	0.0023
	60	4.9	6.4	192.8	0.0029
	70	5	6.4	145.7	0.003
	80	5	6.4	30.4	0.0033
	90	4.9	6.3	15.87	0.0035
CoPBO	0	4.8	63.5	2430	0.00017
	10	5.1	59	2026	0.00035
	20	4.8	52.2	1824.5	0.00033
	30	5.2	36	1789	0.00032

	40	4.9	20.6	1645.8	0.0003
	50	4.8	20.3	1334.7	0.00029
	60	4.8	16.5	1018.8	0.00024
	70	4.8	14.1	745.3	0.00021
CoPBO@Co_3O_4	0	4.9	12.6	1487	0.00043
	10	4.8	12.4	1390	0.00036
	20	5.1	12.3	1135	0.00033
	30	4.8	12.2	862.1	0.00043
	40	5.2	11.9	605.4	0.00073
	50	4.9	11.8	491.7	0.00064
	60	4.8	11.5	407.4	0.001
	70	4.8	11.1	330.9	0.0015

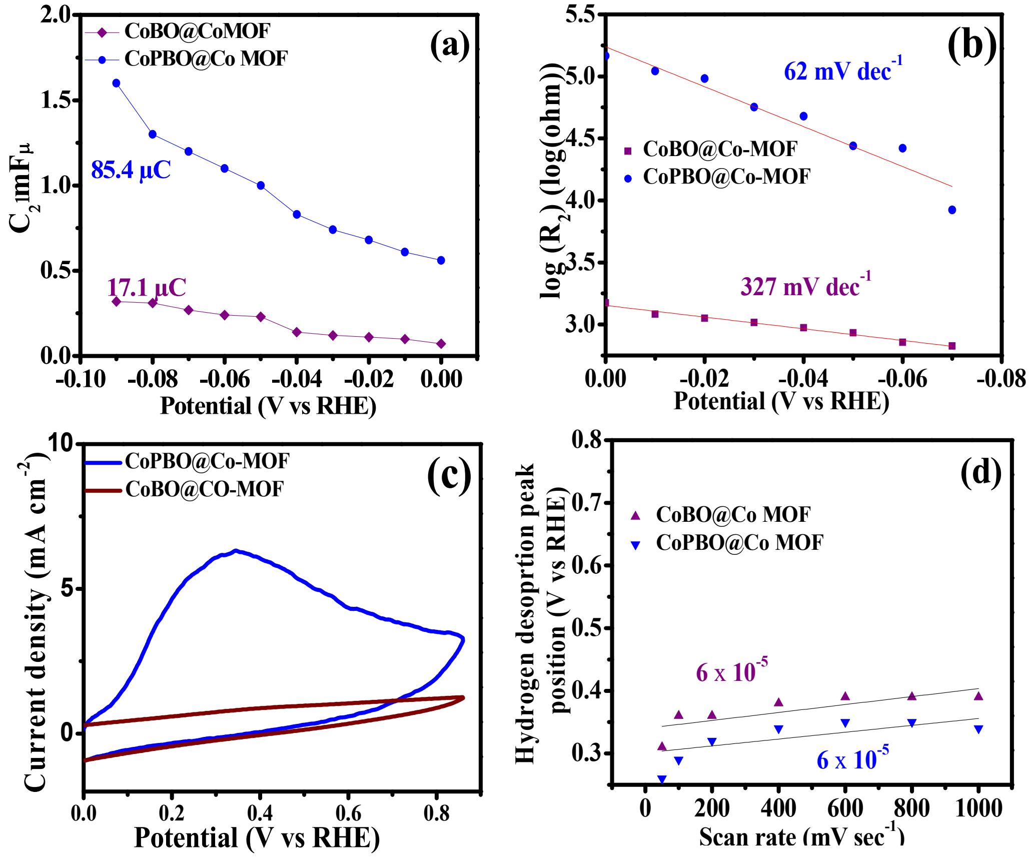

Figure S12: (a) Plot of C_2 vs applied potential, (b) EIS derived Tafel plots, (c) Cyclic voltammetry at the scan rate of 50 mV s^{-1}, and (d) Plots of hydrogen desorption peak position vs scan rate of CoBO@Co-MOF, and CoPBO@Co-MOF for HER.

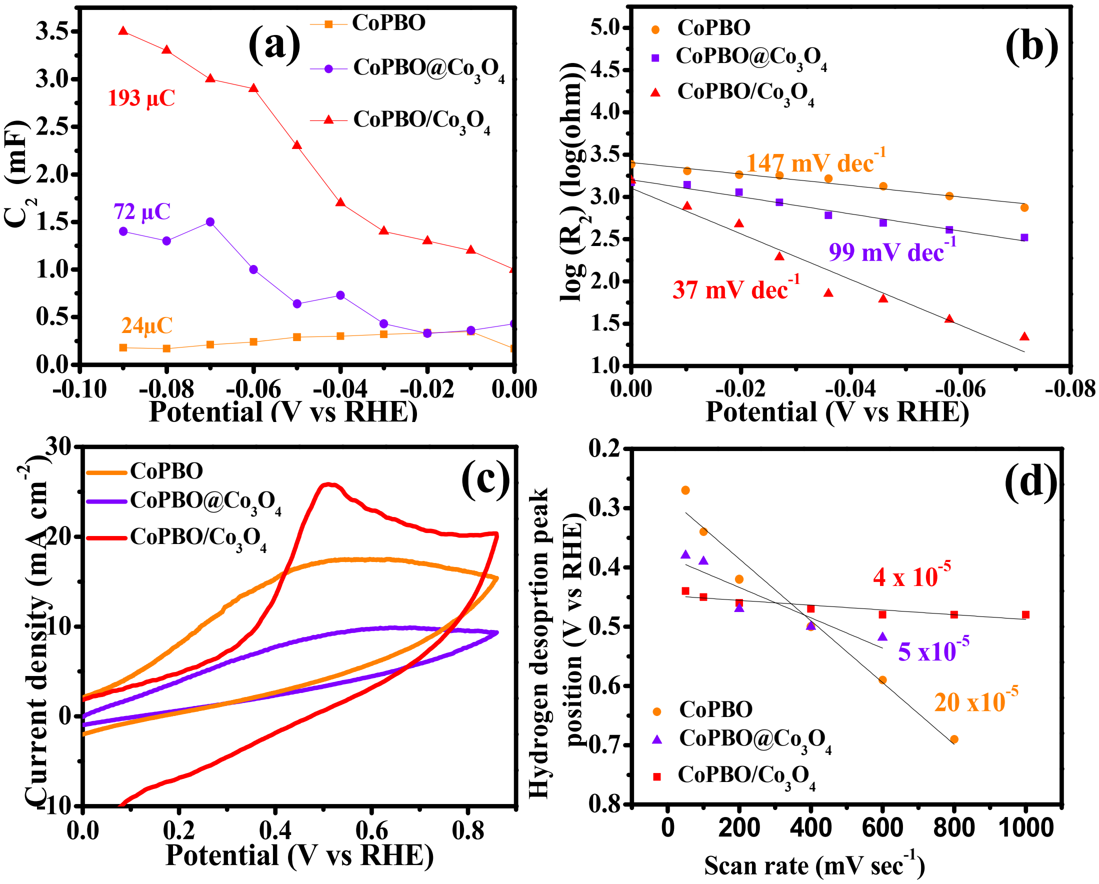

Figure S13: (a) Plot of C_2 vs applied potential, (b) EIS-derived Tafel plots, (c) Cyclic voltammetry at the scan rate of 50 mVs^{-1}, and (d) Plots of hydrogen desorption peak position vs scan rate of CoPBO/Co_3O_4,CoPBO@Co_3O_4, and CoPBO for HER.

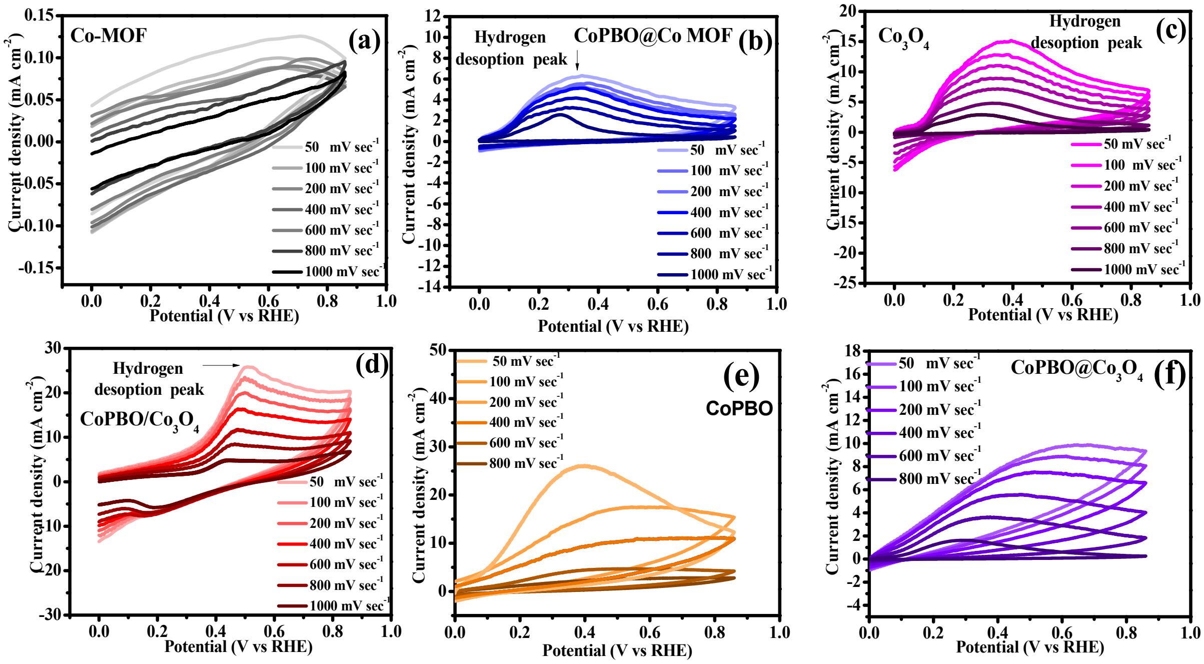

Figure S14: Cyclic voltammetry of (a) Co-MOF, (b) CoPBO@Co-MOF, (c) MOF-derived Co_3O_4, (d) $CoPBO/Co_3O_4$, (e) CoPBO,and(f) $CoPBO@Co_3O_4$,at different scan rates in 1M KOH for HER

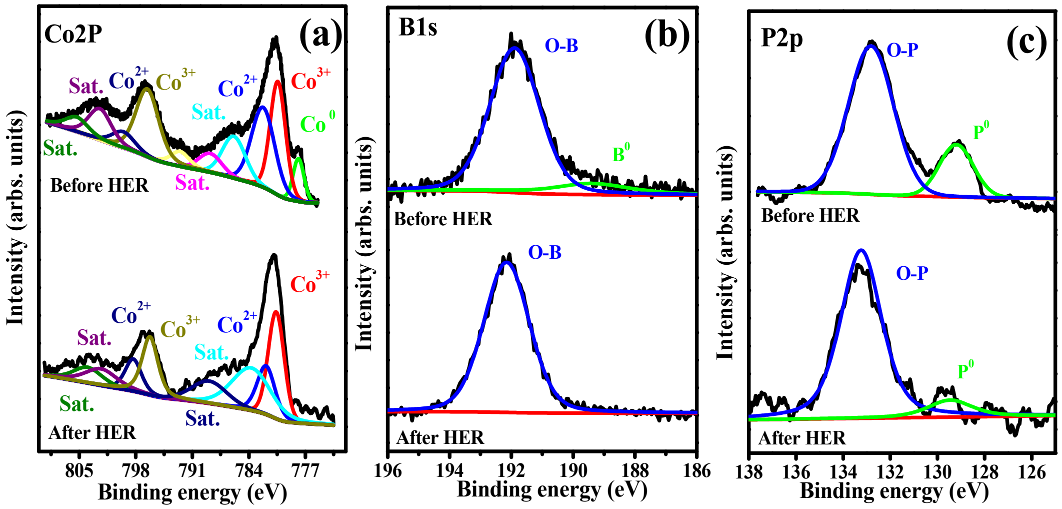

Figure S15: XPS spectra of (a) Co2p, (b) B1s, (c) P2p level of $CoPBO/Co_3O_4$ before and post-HER

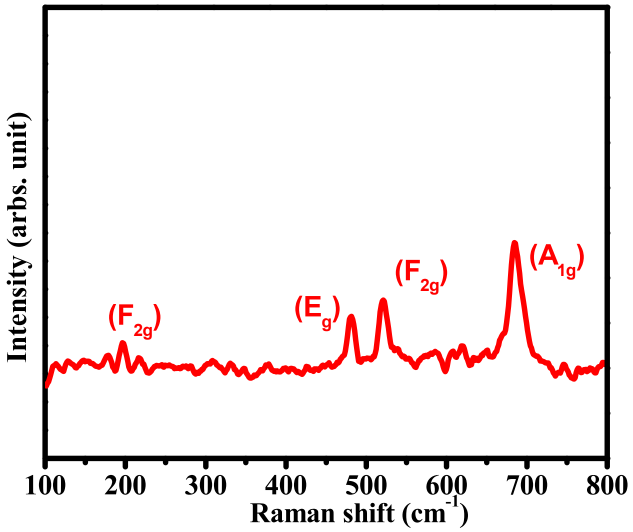

Figure S16: Raman spectra of CoPBO/Co_3O_4post-HER

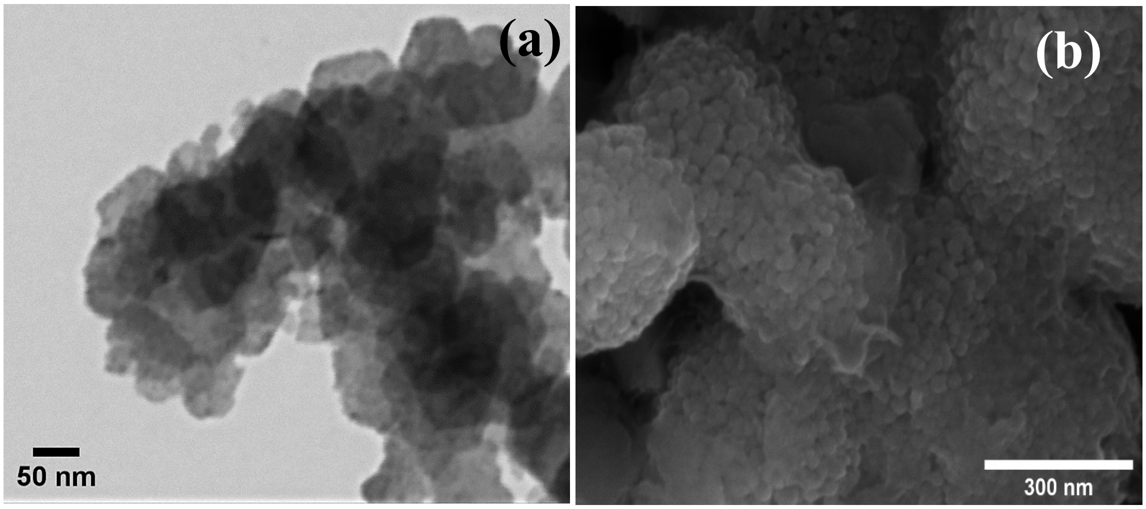

Figure S17: STEM (a) and SEM (b) image of CoPBO/Co_3O_4post-HER

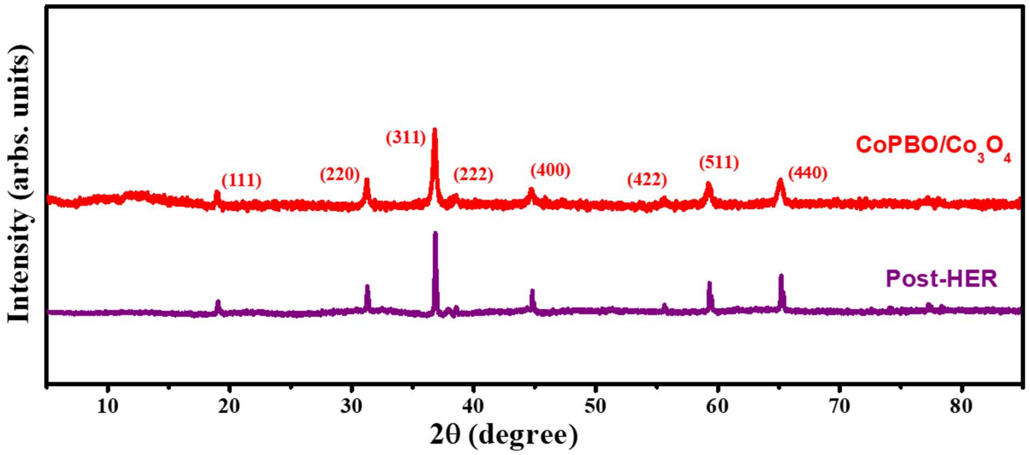

Figure S18: XRD of CoPBO/Co_3O_4post-HER

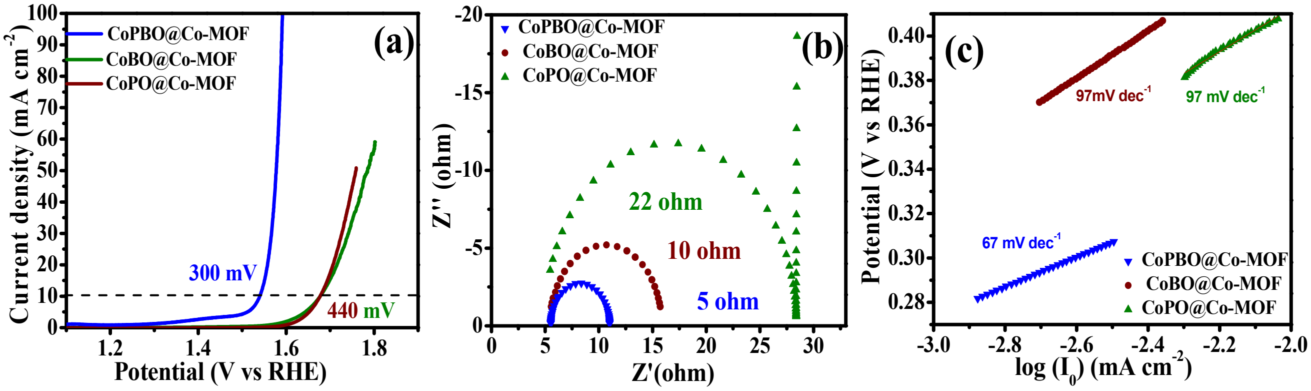

Figure S19:(a) Linear polarization curves, (b) Electrochemical Impedance Spectra,(c) Tafel plot ofCoPO@Co-MOF, CoBO@Co-MOF, and CoPBO@Co-MOF for OER

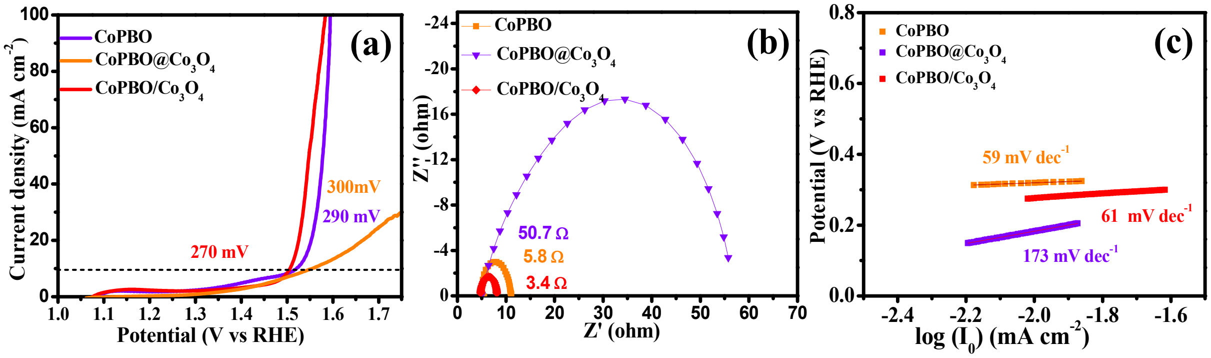

Figure S20: (a) Linear polarization curves, (b) Electrochemical Impedance Spectra, (c) Tafel plot of CoPBO, $CoPBO@Co_3O_4$, and $CoPBO/Co_3O_4$, for OER

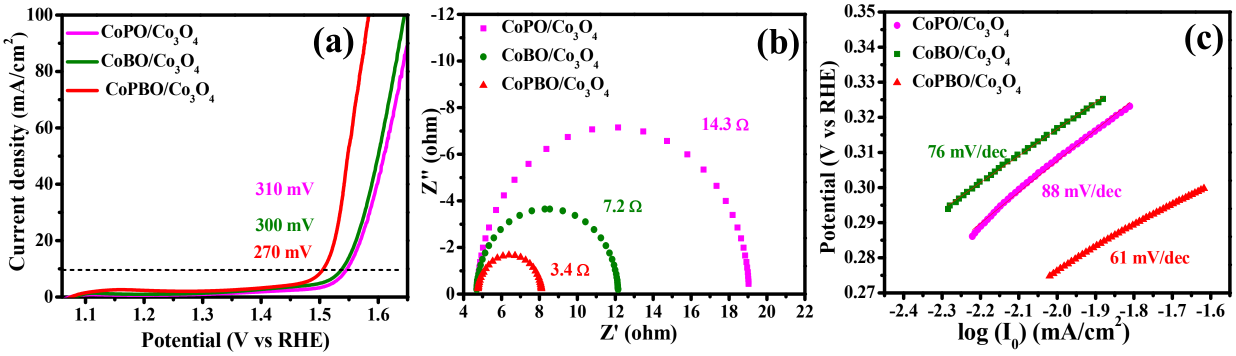

Figure S21: (a) Linear polarization curves, (b) Electrochemical Impedance Spectra, (c) Tafel plot of $CoPO/Co_3O_4$, $CoBO/Co_3O_4$, and $CoPBO/Co_3O_4$, for OER

Supplementary Table S4: Recently reported borides and phosphides-based electrocatalyst performance for OER in alkaline medium.

Electrocatalyst	Overpotential (mV@10mA cm^{-2})	Ref. no.
CoPB	290	[1]
CoWPB	262	[2]
CoWB	292	[3]
Ni-P film/Cu foil	334	[6]
NiFeP	271	[8]
Fe_3Co_7-B/CNT	265	[17]
N_xP_y-T	320	[18]
Co@Co-Bi/Ti	327	[19]
Co_2B-500	380	[20]
$FeCoNiBO_x/PP_y$/rGO	290	[21]
Ni_3B/rGO	290	[22]
Co-B@CoO/Ti	286	[23]
**CoPBO/Co_3O_4**	**270**	**This work**

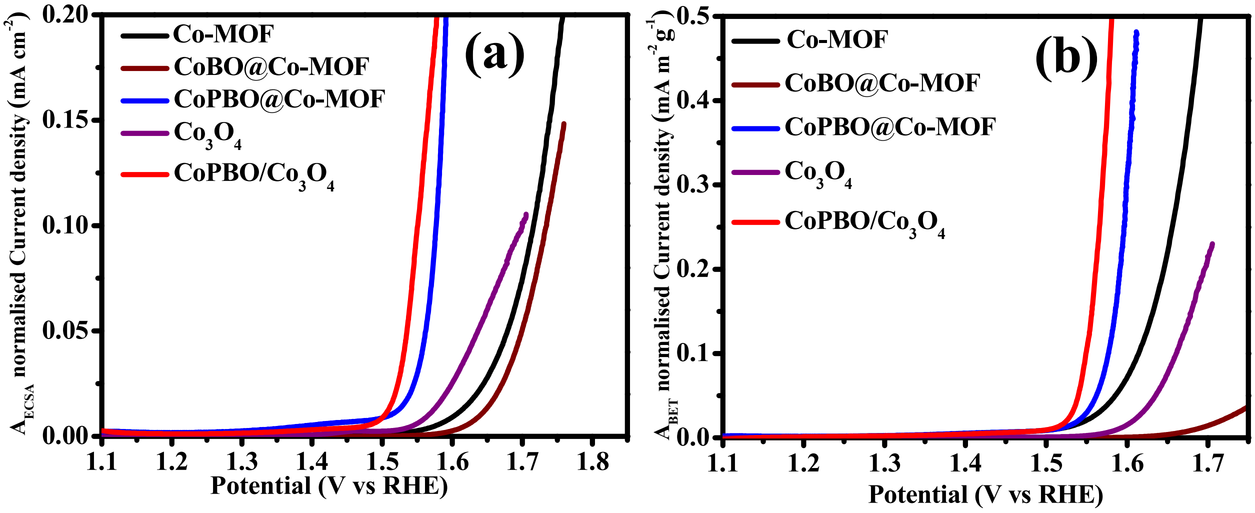

Figure S22: Electrochemical surface area (a) and BET surface area (b) normalized linear sweep voltammograms of Co-MOF, CoBO@Co-MOF, CoPBO@Co-MOF, MOF-derived Co_3O_4, and CoPBO/Co_3O_4 for OER

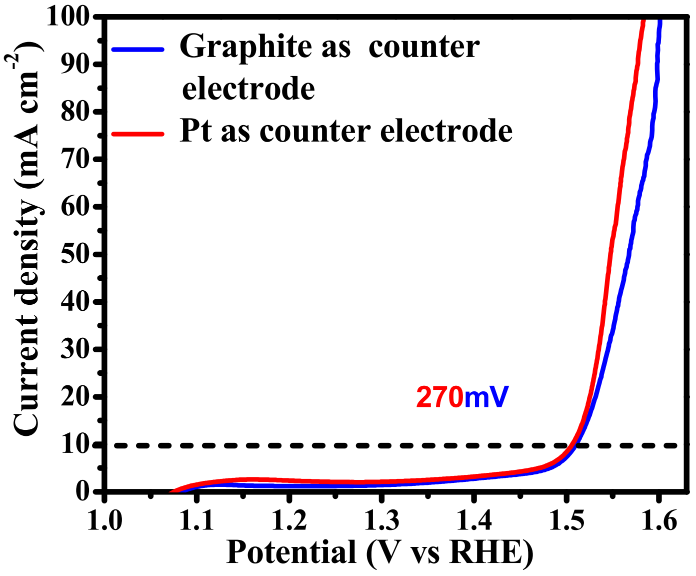

Figure S23:Linear polarization curve of CoPBO/$Co_3O_4$with Pt andgraphite as a counter electrode for OER

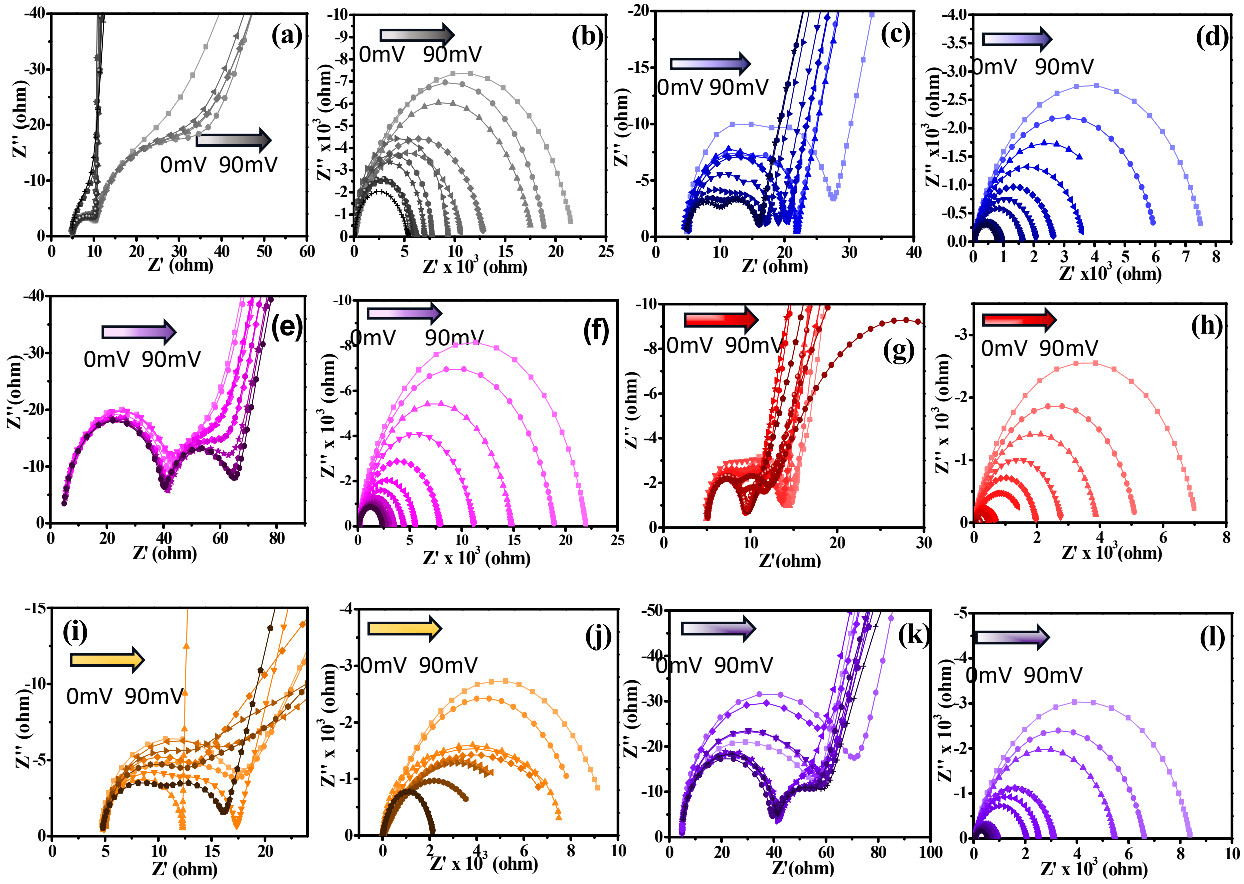

Figure S24:Electrochemical impedance spectra depicting R_{CT} and R_{film} of (a) Co-MOF, (c) CoPBO@Co-MOF, (e) MOF-derived Co_3O_4, (g) CoPBO/Co_3O_4,(i) CoPBO, and (k) CoPBO@Co_3O_4, and R_{OH} of (b) Co-MOF, (d) CoPBO@Co-MOF, (f) MOF-derived Co_3O_4, (h) CoPBO/Co_3O_4, (j) CoPBO, (l) CoPBO@Co_3O_4 at different potentials for OER in 1M KOH

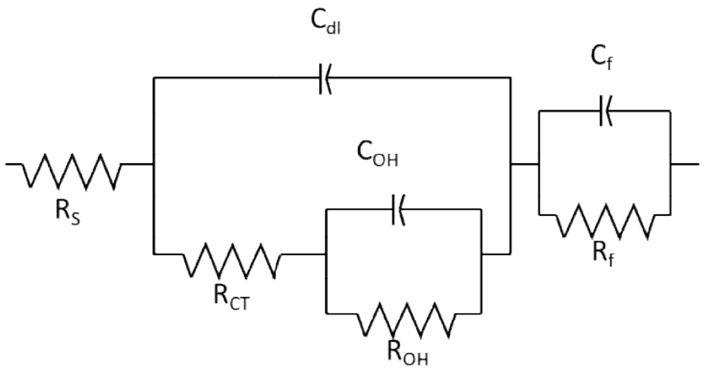

Figure S25: Equivalent circuit utilized for fitting Nyquist Plot for OER.

Supplementary Table S5: The fitted parameters of the EIS data of Co-MOF, CoPBO@Co-MOF, MOF-derived Co_3O_4CoPBO/Co_3O_4, CoPBO, and CoPBO@Co_3O_4 for OER.

Electrocatalyst	Potential V vs RHE	R_S Ω	R_{CT} Ω	R_f Ω	C_{OH} mF	R_{OH} Ω
Co-MOF	1.21	4.9	6.16	26.87	0.16	21874
	1.26	4.8	6.28	26.56	0.19	19005.2
	1.31	4.8	6.21	26.24	0.1	17642
	1.36	4.8	5.98	26.54	0.2	15066.3
	1.41	4.7	5.87	26.13	0.21	12888.2
	1.46	5.9	5.93	24.78	0.22	10997
	1.51	4.8	5.67	23.8	0.23	9234
	1.56	4.9	5.86	23.79	0.24	7696
	1.61	4.8	5.92	23.98	0.28	6997
	1.67	4.5	5.64	25.78	0.45	6210
CoPBO@co-MOF	1.21	4.6	20.07	17.86	0.27	7588
	1.26	4.8	19.75	16.65	0.43	6001
	1.31	4.8	18.35	15.85	0.54	4788
	1.36	4.9	18.14	14.87	0.65	3638
	1.41	4.8	17.54	14.47	0.68	2657
	1.46	5.1	15.7	13.8	1.1	2072
	1.51	4.8	13.86	11.7	1.4	1609
	1.56	5.2	11.98	9.64	2.9	1165
	1.61	4.8	9.76	7.65	4.5	961
	1.67	5.3	7.25	6.76	6.7	801
Co_3O_4	1.21	5.1	37.76	44.6	0.11	21939
	1.26	4.9	37.53	43.73	0.12	18891
	1.31	4.8	37.86	42.87	0.5	14782
	1.36	5.1	37.65	41.33	0.54	11122
	1.41	4.9	36.45	41.12	0.57	7877
	1.46	5.2	36.98	40.73	0.87	5528
	1.51	4.8	36.28	37.81	0.83	4337
	1.56	5.2	36.83	35.82	0.93	3444
	1.61	4.7	36.97	30.65	1	2984
	1.67	5.1	36.35	26.62	1.1	2652

CoPBO/Co_3O_4	1.21	4.9	4.12	4.76	0.48	7052
	1.26	4.8	3.95	4.63	1.00114	5140
	1.31	4.8	3.75	4.47	0.85129	3904
	1.36	5.2	3.65	4.15	1.75519	2768
	1.41	5.1	3.63	3.92	3.07478	1974
	1.46	4.9	3.58	3.81	4.86807	1185

	1.51	4.8	3.52	3.63	5.91697	797
	1.56	4.8	3.49	3.58	7.55075	580
	1.61	4.9	3.47	3.28	9.8	467
	1.67	5.1	3.4	2.89	14.6	330
CoPBO	1.21	5	8.17	--	0.21	9764
	1.26	4.9	8.38	--	0.3	8653
	1.31	5	7.17	--	0.47	7456
	1.36	4.9	7.64	9.63	0.5	6653
	1.41	5	7.96	9.74	0.55	6432
	1.46	4.9	7.59	9.52	0.62	6174
	1.51	5.1	7.52	9.1	0.85	4372
	1.56	4.9	8.17	8.63	1.6	4198
	1.61	5	6.73	6.95	2.6	3184
	1.67	4.9	5.86	6.74	3.7	2673
CoPBO@Co_3O_4	1.21	5	36.16	--	0.24	8352
	1.26	4.9	36.12	--	0.25	6553
	1.31	5	36.4	--	0.31	5428
	1.36	4.9	36.2	--	0.35	4235
	1.41	4.9	36.27	--	0.41	3047
	1.46	5	36.17	20.86	0.41	2487
	1.51	5	36.4	20.42	0.72	1968
	1.56	5	36.13	19.83	0.74	1433
	1.61	4.9	36.35	19.67	1.1	1137
	1.67	5	36.26	19.65	1.9	889

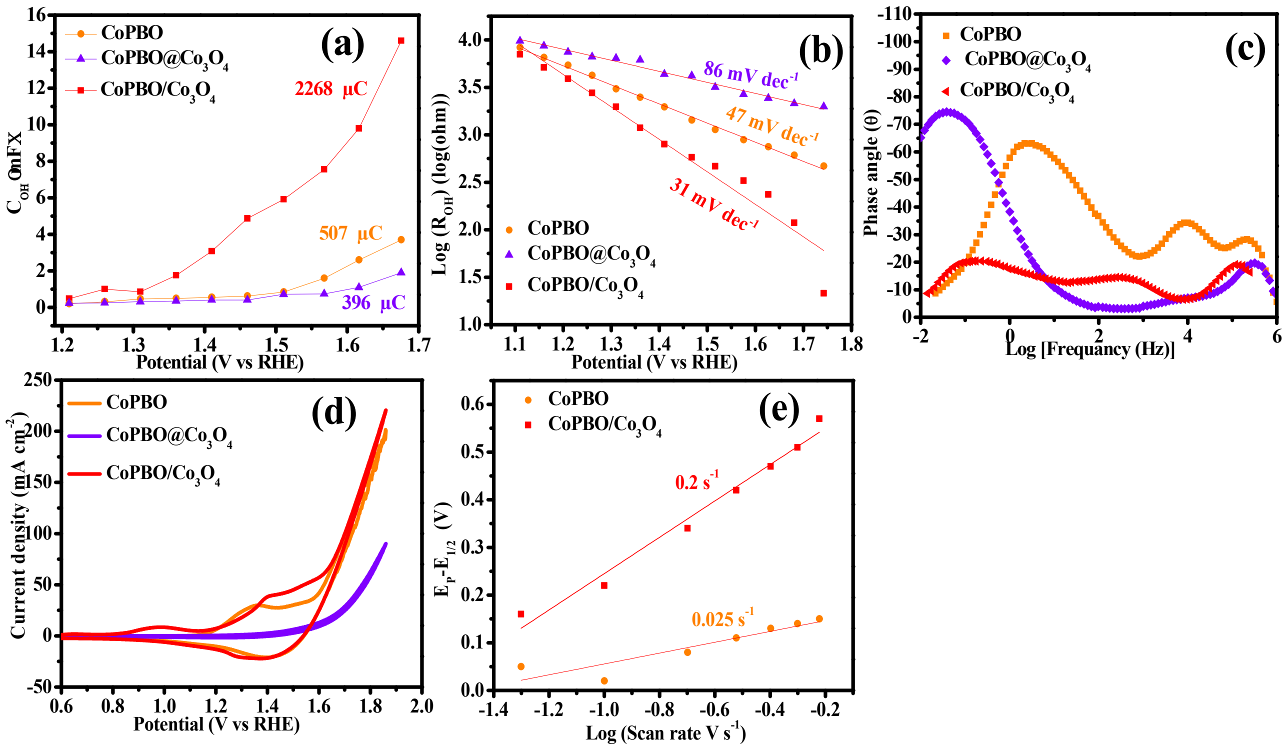

Figure S26:(a) Plot of C_{OH} vs applied potential, (b) EIS derived Tafel plots, (c) Bode plot acquired at 1.5 V (vs RHE), (d) Cyclic voltammetry at scan rate of 50 mV/sec with inset showing magnified CV plot of Co_3O_4, and (e) plot of the difference between the redox peak of Co^{3+}/Co^{4+} vs. log of scan rate for OER of CoPBO,CoPBO@ Co_3O_4, and CoPBO/Co_3O_4, for OER

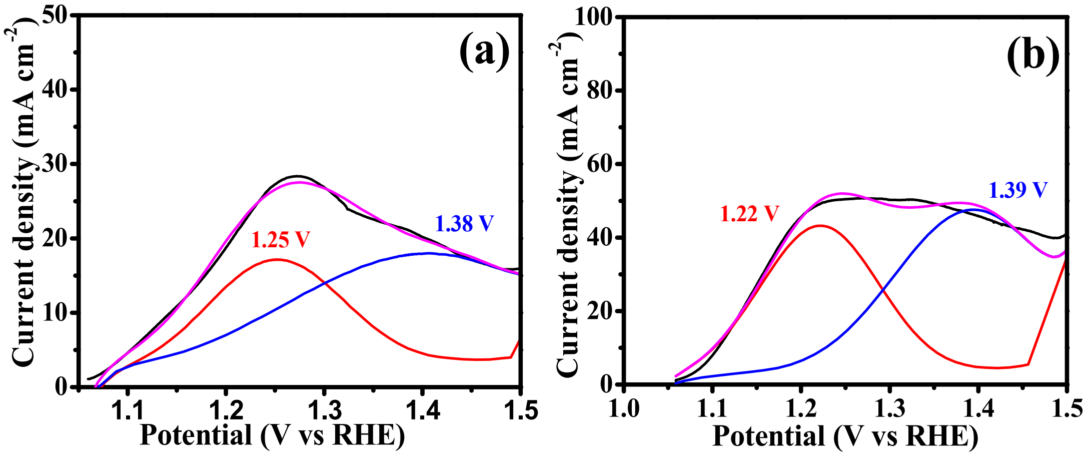

Figure S27: Deconvoluted pre-oxidation peaks of (a) CoPBO@Co-MOF and (b) CoPBO/Co_3O_4 for OER

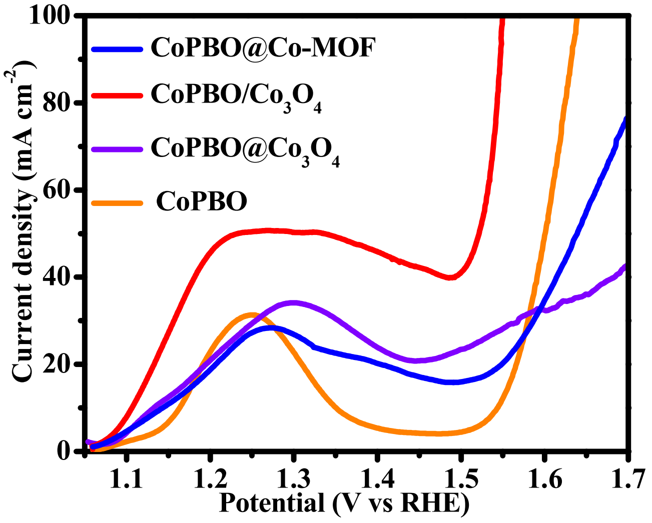

Figure S28: LSV in the pre-OER potential region acquired at a scan rate of 2 mV s^{-1} of CoPBO@Co-MOF, CoPBO/Co_3O_4, CoPBO@Co_3O_4, and CoPBO electrocatalyst.

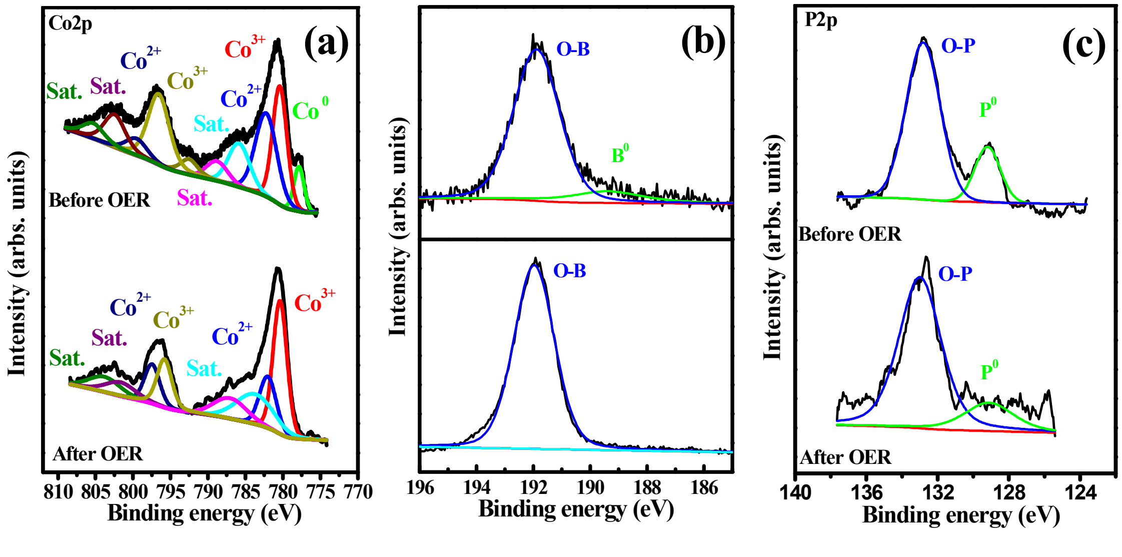

Figure S29: XPS spectra in (a) Co2p, (b) B1s, (c) P2p level of CoPBO/Co_3O_4 before and post-OER

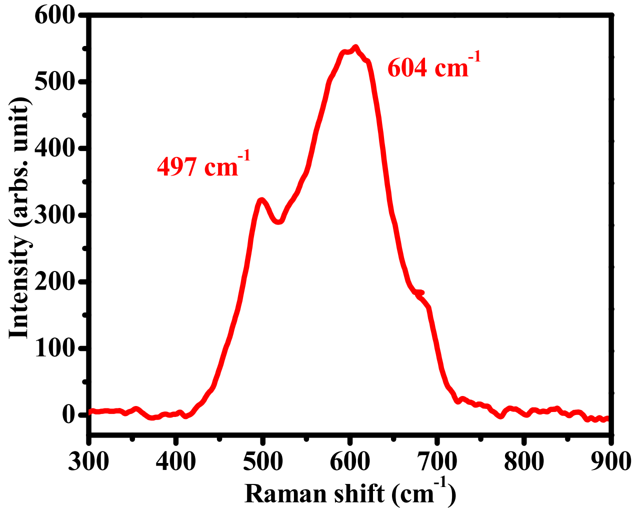

Figure S30: Raman spectra of CoPBO/Co_3O_4post-OER

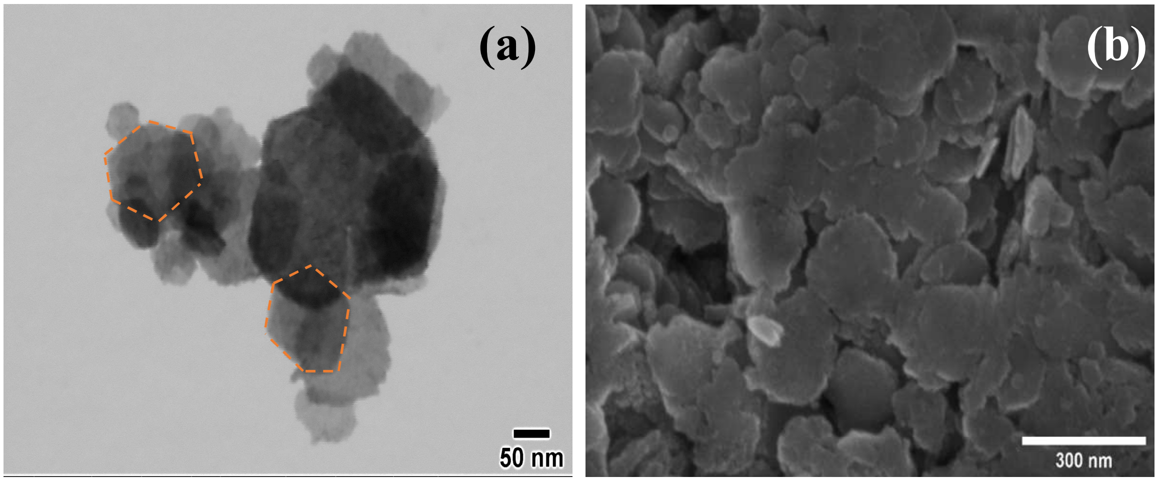

Figure S31: STEM (a) and SEM (b) image of $CoPBO/Co_3O_4$ post-OER

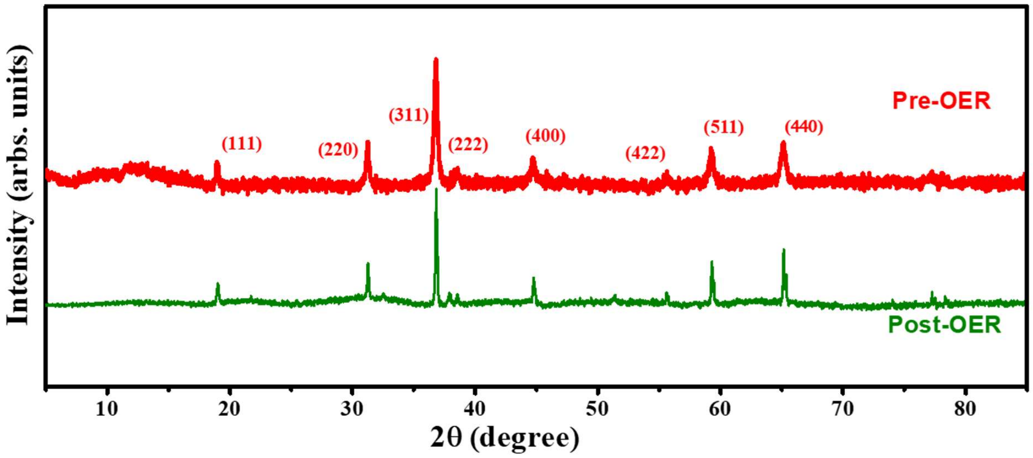

Figure S32: XRD of $CoPBO/Co_3O_4$ post-OER

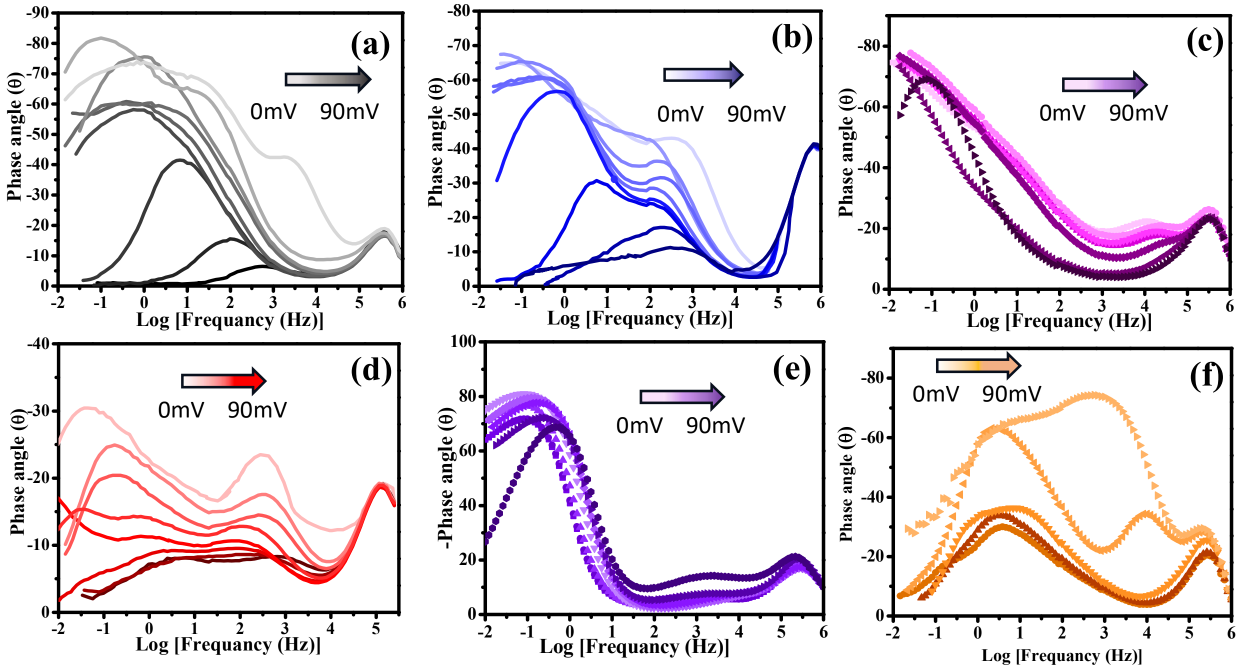

Figure S33: Bode plot of (a) Co-MOF, (b) CoPBO@Co-MOF, (c) MOF-derived Co_3O_4, (d) CoPBO/Co_3O_4,(e) CoPBO@Co_3O_4, and (f) CoPBO at different potentials for OER in 1M

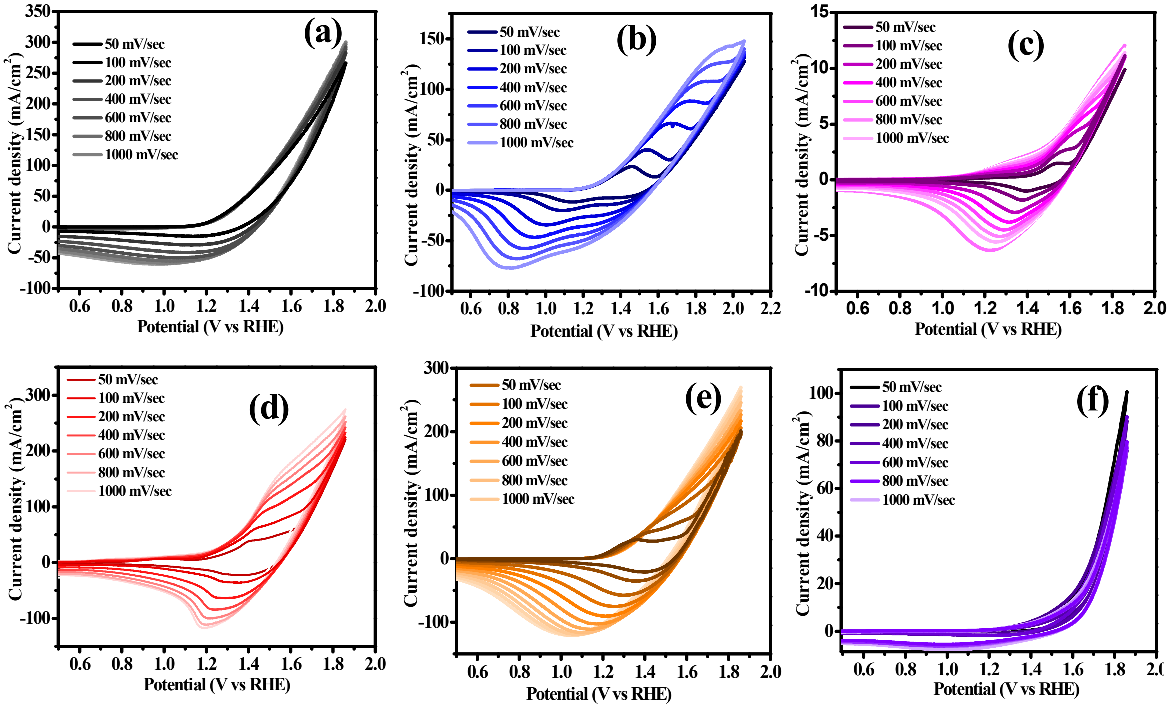

Figure S34: Cyclic voltammetry of (a) Co-MOF, (b) CoPBO@Co-MOF, (c) MOF-dervied Co_3O_4,(d) CoPBO/Co_3O_4,(e) CoPBO, and (f)CoPBO@ Co_3O_4at different scan rates for OER in 1M KOH

Supplementary Table S6: Overall water splitting performances of recently reported bifunctional electrocatalysts @10 mA/cm2 inatwo-electrode setup using glassy carbon electrode as support in 1M KOH.

**Electrocatalyst**	**Cell Voltage**	**References**
$CoPBO/Co_3O_4$	1.59	**This work**
Co-BTC	2.03	[24]
NiCoZnP/NC	1.62	[25]
$Co_{0.85}Se$@NC	1.76	[26]
Co-NC/CNT	1.76	[27]
$Ni_3ZnC_{0.7}$	1.65	[28]
Ni@NV-800/NF	1.6	[29]
$Co/CoN/Co_2P$-NPC	1.6	[30]
Ni-Co-S HPNA	1.62	[31]
RuO_2/Co_3O_4	1.64	[32]
NiCoFeB	1.81	[33]

References:

[1] A. Chunduri, S. Gupta, O. Bapat, A. Bhide, R. Fernandes, M. K. Patel, V. Bambole, *Appl. Catal. B Environ.* **2019**, *259*, 118051.

[2] A. Bhide, S. Gupta, R. Bhabal, K. H. Mali, B. R. Bhagat, A. Dashora, M. Patel, R. Fernandes, N. Patel, *Int. J. Hydrogen Energy* **2024**, *63*, 645.

[3] A. Chunduri, A. Bhide, S. Gupta, K. H. Mali, B. R. Bhagat, A. Dashora, M. Spreitzer, R. Fernandes, R. Patel, N. Patel, *ACS Appl. Energy Mater.* **2023**, *6*, 4630.

[4] F. Paquin, J. Rivnay, A. Salleo, N. Stingelin, C. Silva, *J. Mater. Chem. C* **2015**, *3*, 10715.

[5] Z. Wu, D. Nie, M. Song, T. Jiao, G. Fu, X. Liu, *Nanoscale* **2019**, *11*, 7506.

[6] N. Jiang, B. You, M. Sheng, Y. Sun, *ChemCatChem* **2016**, *8*, 106.

[7] L. Yan, H. Jiang, Y. Xing, Y. Wang, D. Liu, X. Gu, P. Dai, L. Li, X. Zhao, *J. Mater. Chem. A* **2018**, *6*, 1682.

[8] C. Xuan, J. Wang, W. Xia, Z. Peng, Z. Wu, W. Lei, K. Xia, H. L. Xin, D. Wang, *ACS Appl. Mater. Interfaces* **2017**, *9*, 26134.

[9] K. Wang, Y. Si, Z. Lv, T. Yu, X. Liu, G. Wang, G. Xie, L. Jiang, *Int. J. Hydrogen Energy* **2020**, *45*, 2504.

[10] F. Yang, S. Huang, B. Zhang, L. Hou, Y. Ding, W. Bao, C. Xu, W. Yang, Y. Li, *Nanomaterials* **2019**, *9*, DOI 10.3390/nano9071022.

[11] M. Miao, R. Hou, Z. Liang, R. Qi, T. He, Y. Yan, K. Qi, H. Liu, G. Feng, B. Y. Xia, *J. Mater. Chem. A* **2018**, *6*, 24107.

[12] Z. Pu, C. Zhang, I. S. Amiinu, W. Li, L. Wu, S. Mu, Z. Pu, C. Zhang, I. S. Amiinu, W. Li, L. Wu, S. Mu, **2017**.

[13] H. Qu, Y. Ma, Z. Gou, B. Li, Y. Liu, Z. Zhang, L. Wang, *J. Colloid Interface Sci.* **2020**, *572*, 83.

[14] Y. Wei, P. Zou, Y. Yue, M. Wang, W. Fu, S. Si, L. Wei, X. Zhao, G. Hu, H. L. Xin, *ACS Appl. Mater. Interfaces* **2021**, *13*, 20024.

[15] C. B. Sun, M. W. Guo, S. S. Siwal, Q. B. Zhang, *J. Catal.* **2020**, *381*, 454.

[16] D. Dai, B. Wei, Y. Li, X. Ma, S. Liang, S. Wang, L. Xu, *J. Alloys Compd.* **2020**, *820*, 153185.

[17] J. M. V. Nsanzimana, L. Gong, R. Dangol, V. Reddu, V. Jose, B. Y. Xia, Q. Yan, J. M. Lee, X. Wang, *Adv. Energy Mater.* **2019**, *9*, 1.

[18] J. Li, J. Li, X. Zhou, Z. Xia, W. Gao, Y. Ma, Y. Qu, *ACS Appl. Mater. Interfaces* **2016**, *8*, 10826.

[19] C. Xie, Y. Wang, D. Yan, L. Tao, S. Wang, *Nanoscale* **2017**, *9*, 16059.

[20] J. Masa, P. Weide, D. Peeters, I. Sinev, W. Xia, Z. Sun, C. Somsen, M. Muhler, W. Schuhmann, *Adv. Energy Mater.* **2016**, *6*, 1.

[21] H. Mao, X. Guo, Y. Fu, H. Yang, Y. Zhang, R. Zhang, X. M. Song, *J. Mater. Chem. A* **2020**, *8*, 1821.

[22] M. Arivu, J. Masud, S. Umapathi, M. Nath, *Electrochem. commun.* **2018**, *86*, 121.

[23] W. Lu, T. Liu, L. Xie, C. Tang, D. Liu, S. Hao, F. Qu, G. Du, Y. Ma, A. M. Asiri, X. Sun, *Small* **2017**, *13*, 1.

[24] S. Naik Shreyanka, J. Theerthagiri, S. J. Lee, Y. Yu, M. Y. Choi, *Chem. Eng. J.***2022**, *446*, 137045.

[25] B. Chen, D. Kim, Z. Zhang, M. Lee, K. Yong, *Chem. Eng. J.***2021**, *422*, 130533.

[26] T. Meng, J. Qin, S. Wang, D. Zhao, B. Mao, M. Cao, *J. Mater. Chem. A***2017**, *5*, 7001.

[27] F. Yang, P. Zhao, X. Hua, W. Luo, G. Cheng, W. Xing, S. Chen, *J. Mater. Chem. A***2016**, *4*, 16057.

[28] Y. Wang, W. Wu, Y. Rao, Z. Li, N. Tsubaki, M. Wu, *J. Mater. Chem. A***2017**, *5*, 6170.

[29] Y. Xu, W. Tu, B. Zhang, S. Yin, Y. Huang, M. Kraft, R. Xu, *Adv. Mater.***2017**, *29*, 1.

[30] L. Hu, Y. Hu, R. Liu, Y. Mao, M. S. (Jie T. Balogun, Y. Tong, *Int. J. Hydrogen Energy***2019**, *44*, 11402.

[31] W. Chen, Y. Zhang, G. Chen, R. Huang, Y. Wu, Y. Zhou, Y. Hu, K. (Ken) Ostrikov, *J. Colloid Interface Sci.***2020**, *560*, 426.

[32] H. Liu, G. Xia, R. Zhang, P. Jiang, J. Chen, Q. Chen, *RSC Adv.***2017**, *7*, 3686.

[33] Y. Li, B. Huang, Y. Sun, M. Luo, Y. Yang, Y. Qin, L. Wang, C. Li, F. Lv, W. Zhang, S. Guo, *Small***2019**, *15*, 1.
